# DNA methylation in AgRP neurons regulates voluntary exercise behavior in mice

**DOI:** 10.1038/s41467-019-13339-3

**Published:** 2019-12-02

**Authors:** Harry MacKay, C. Anthony Scott, Jack D. Duryea, Maria S. Baker, Eleonora Laritsky, Amanda E. Elson, Theodore Garland Jr., Marta L. Fiorotto, Rui Chen, Yumei Li, Cristian Coarfa, Richard B. Simerly, Robert A. Waterland

**Affiliations:** 1 Department of Pediatrics, Baylor College of Medicine, USDA/ARS Children’s Nutrition Research Center, Houston, TX 77030 USA; 2 Department of Molecular Physiology & Biophysics, Vanderbilt University, Nashville, TN 37235 USA; 3 Department of Evolution, Ecology, and Organismal Biology, University of California, Riverside, CA 92521 USA; 4 Department of Molecular Physiology & Biophysics, Baylor College of Medicine, Houston, TX 77030 USA; 5 Department of Molecular & Human Genetics, Baylor College of Medicine, Houston, TX 77030 USA; 6 Department of Molecular & Cell Biology, Baylor College of Medicine, Houston, TX 77030 USA

**Keywords:** Epigenetics and behaviour, Hypothalamus

## Abstract

DNA methylation regulates cell type-specific gene expression. Here, in a transgenic mouse model, we show that deletion of the gene encoding DNA methyltransferase Dnmt3a in hypothalamic AgRP neurons causes a sedentary phenotype characterized by reduced voluntary exercise and increased adiposity. Whole-genome bisulfite sequencing (WGBS) and transcriptional profiling in neuronal nuclei from the arcuate nucleus of the hypothalamus (ARH) reveal differentially methylated genomic regions and reduced expression of AgRP neuron-associated genes in knockout mice. We use read-level analysis of WGBS data to infer putative ARH neural cell types affected by the knockout, and to localize promoter hypomethylation and increased expression of the growth factor Bmp7 to AgRP neurons, suggesting a role for aberrant TGF-β signaling in the development of this phenotype. Together, these data demonstrate that DNA methylation in AgRP neurons is required for their normal epigenetic development and neuron-specific gene expression profiles, and regulates voluntary exercise behavior.

## Abstract (web summary)

AgRP neurons in the hypothalamic arcuate nucleus (ARH) are involved in regulating hunger and energy balance. Here the authors show that knockout of the DNA methyltransferase Dnmt3a in AgRP neurons of the ARH leads to a reduction in voluntary exercise along with numerous epigenetic and gene expression changes in ARH neurons.

## Introduction

Homeostatic regulation of body weight involves a balance between energy intake and expenditure. The expenditure side of the equation is dominated by basal metabolism in sedentary individuals, but also includes voluntary exercise and other forms of physical activity^1^. The roots of the obesity epidemic are multifactorial, but physical inactivity is a significant factor and itself a substantial contributor to worldwide morbidity and mortality^2^. Understanding the origin of interindividual differences in physical activity is therefore essential. Whereas sociocultural environment and genetic variation^3^ clearly play an important role, mouse models of developmental programming show that environmental influences during critical ontogenic periods also have a substantial impact on adult voluntary physical activity^4–6^.

Our current understanding of physiological mechanisms regulating voluntary physical activity is rudimentary^3^. Recent studies, however, suggest a link between hormonal signals of energy balance such as leptin and locomotor activity^7^. In particular, AgRP neurons in the hypothalamic arcuate nucleus (ARH), conventionally seen as the orexigenic side of the melanocortin system^8^, also influence locomotor activity^9–12^, and restoration of leptin receptor activity in the ARH is sufficient to rescue home cage locomotor activity in *Lepr* KO mice^13^.

The epigenetic mechanism DNA methylation, established in neurons during the perinatal period by the de novo DNA methyltransferase *Dnmt3a*^14^, plays a fundamental role in neuronal differentiation^15^. We previously reported widespread increases in CpG methylation in mouse hypothalamic neurons from postnatal day 0 (P0) to P21^16^—particularly at neurodevelopmental genes—suggesting that DNA methylation plays an active role in hypothalamic development by epigenetically silencing genes at developmentally appropriate timepoints. Accordingly, we hypothesized that perturbing the establishment of DNA methylation in the hypothalamus will disrupt cellular differentiation and energy homeostasis. Previous studies of hypothalamic neuroepigenetics, using similar logic, provide evidence that DNA methylation plays a role in sexual differentiation^17^, circadian rhythms^18^, and food intake^19^. However, as those studies relied on local injection of methyltransferase inhibitors^17,18^ or non-cell type-specific knockout of *Dnmt3a*^17,19^, it remains unclear to what extent epigenetic changes within specific neuronal cell types can affect energy balance. Indeed, a major current limitation in neuroepigenetics is the difficulty of linking cell type-specific epigenetic changes to specific adult phenotypes.

Here we show that AgRP neuron-specific knockout of *Dnmt3a* leads to cell type-specific disruption of DNA methylation and developmental gene expression, culminating in a lower physical activity set point. Moreover, our epigenomic analyses indicate that AgRP neuron-specific changes in DNA methylation at *Bmp7* increase the expression of this paracrine signaling molecule, leading to widespread effects on TGF-β signaling in the arcuate nucleus. Our findings demonstrate a crucial role for DNA methylation in the normal development of the hypothalamic energy balance circuitry and indicate that epigenetic mechanisms established early in life regulate individual proclivity for physical activity.

## Results

### Dnmt3a regulates DNA methylation in AgRP neurons

**Fig. 1:**
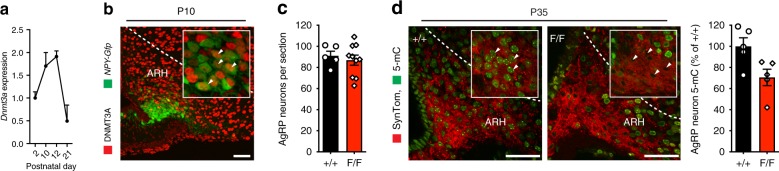
AgRP neuron-specific knockout of Dnmt3a reduces DNA methylation in AgRP neurons. **a** Dnmt3a expression peaks in the postnatal ARH at P12 (*n* = 5–8 per timepoint). **b** Immunostaining for Dnmt3a shows that AgRP/NPY neurons (labeled by GFP) express Dnmt3a at P10 (inset: 63× confocal image, NPY+; DNMT3A+ neurons indicated by arrow). Scale = 10 μm. **c** AgRP neuron-specific Dnmt3a knockout does not affect AgRP neuronal density, *t*(13) = 0.55, *p* = 0.59, *n* = 5–8. **d** Relative to +*/*+ mice, AgRP neurons (labeled by SynTom) of *F/F* mice show reduced levels of 5-methylcytosine; left—representative immunofluorescent labeling of 5-mC in SynTom+ AgRP neurons (inset: 63× confocal image, representative AgRP neurons indicated by arrow), right—quantitation of 5-mC labeling intensity in AgRP neurons, *t*(8) = 2.64, *p* = 0.03, *n* = 5. Scale = 10 μm. Values reflect mean ± SEM. *p < 0.05. Source data for **a, c, d** are provided as a Source Data file.

Because de novo DNA methylation in neurons is regulated by *Dnmt3a*, we first studied the dynamics of *Dnmt3a* expression in the wild-type mouse ARH. In line with findings in other brain regions^14^, *Dnmt3a* expression in the postnatal ARH reached a peak at P12 and declined substantially by P21 (Fig. 1a). We next studied *Dnmt3a* expression by immunofluorescent labeling of Dnmt3a in AgRP/NPY cells identified by the NPY-hrGFP transgene and found substantial co-localization at P10 (Fig. 1b), confirming that AgRP neurons express *Dnmt3a* during postnatal life. To assess the importance of *Dnmt3a* expression in establishing DNA methylation patterns within AgRP neurons, we generated AgRP neuron-specific *Dnmt3a* knockout mice (*AgRP-IRES-Cre*^*+*^; *Dnmt3a*^*F/F*^, hereafter referred to as *F/F* mice) by crossing Agrp^tm1(cre)Lowl^/J mice (see Methods; Supplementary Fig. 1A) with mice harboring loxP sites flanking exon 18 of *Dnmt3a* (see Methods; Supplementary Fig. 1B, C). (*AgRP-IRES-Cre*^*+*^; *Dnmt3a*^*+/+*^ carrying the wild-type *Dnmt3a* allele were used as controls—hereafter referred to as +*/*+ mice). Abrogation of *Dnmt3a* expression did not alter the number of AgRP neurons (Fig. 1c), but did significantly reduce levels of 5-methylcytosine (Fig. 1d). Bisulfite treatment-based sequencing approaches cannot differentiate 5-methylcytosine and the product of TET-mediated demethylation 5-hydroxymethylcytosine^20^. We used immunofluorescent labeling and found that 5-hydroxymethylcytosine was also reduced in putative AgRP neurons (Supplementary Fig. 1D), consistent with the reduction in 5-methylcytosine. These data indicate that *Dnmt3a* helps establish DNA methylation in AgRP neurons.

### Sedentary phenotype in mice lacking *Dnmt3a* in AgRP neurons

**Fig. 2:**
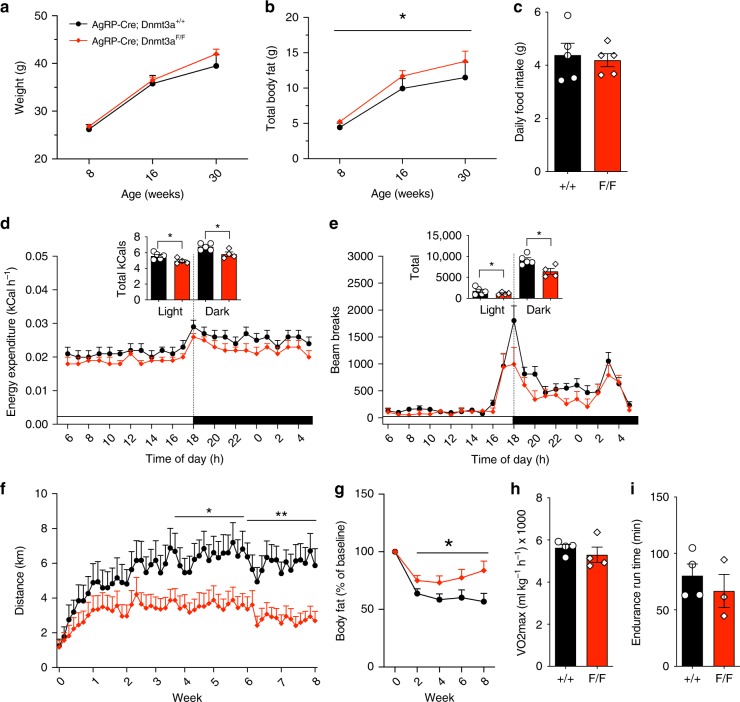
Sedentary phenotype in *F/F* mice. **a** Male *F/F* mice show no difference in body weight relative to +*/*+ mice, *F*(1, 32) = 3.56 Time × Genotype, *p* = 0.068, *n* = 6–12. **b** Male *F/F* mice show increased adiposity *F*(1, 33) = 4.75 main effect of Genotype, *p* = 0.036, *n* = 6–13. **c** Male *F/F* mice show no difference in daily food intake, whether adjusted or unadjusted (not shown) for lean and fat body mass, *F*(1, 6) = 0.003 main effect of Genotype, *p* = 0.955, *n* = 5. **d** Reduced energy expenditure in *F/F* mice. Inset: total caloric expenditure in light and dark periods, *F*(1, 15) = 7.649 Genotype × Sex interaction, *p* = 0.014, post-hoc comparison in male mice *p* = 0.042, *n* = 4–5. **e** Reduced locomotor activity in *F/F* mice. Inset: total locomotion in light and dark periods, *F*(1, 7) = 8.359, *p* = 0.023 main effect of Genotype, *n* = 4–5. **f** When given free access to a running wheel, *F/F* mice ran about half as much as +*/*+ mice (data represent animals from two independent experiments, *F*(3, 72) = 7.258 Time × Genotype, *p* = 0.009). **g** When given access to running wheels, *F/F* mice lose less body fat than +*/*+ mice, *F*(4, 96) = 3.60, *p* = 0.009 Genotype × Time, *F*(1, 24) = 4.77, *p* = 0.0039 main effect of Genotype, *n* = 13. **h** and **i** Metabolic treadmill testing shows that VO_2_Max (**h**), *t*(6) = 0.83, *p* = 0.436, and endurance run time at 60% of VO_2_Max (**i**), *t*(5) = 0.781, *p* = 0.47, do not differ between *F/F* and +*/*+ mice, *n* = 3–4. Values reflect mean ± SEM. ***p < 0.001, **p < 0.01, *p < 0.05. Source data for **a**–**i** are provided as a Source Data file.

Given the central role of AgRP neurons in energy balance homeostasis, we were surprised that there was merely a non-significant trend toward higher body weight in *F/F* adults of both sexes (Fig. 2a; Supplementary Fig. 1G). This was not attributable to a difference in lean body mass (Supplementary Fig. 1E, F); however, *F/F* mice of both sexes did exhibit significantly increased body fat (Fig. 2b; Supplementary Fig. 1H). To probe the cause of the increased adiposity, we performed indirect calorimetry in adult male mice and found that lean- and fat mass-adjusted food intake was unchanged (Fig. 2c) but lean- and fat mass-adjusted energy expenditure was reduced in *F/F* mice (Fig. 2d). Since resting metabolic rate did not differ between genotypes (Supplementary Fig. 1I), this deficit is specific to the non-resting component (Supplementary Fig. 1J), consistent with reduced home cage activity in *F/F* mice (Fig. 2e). The cages used for indirect calorimetry offer limited space for physical activity, so we next offered an independent cohort of adult mice free access to running wheels for 8 weeks. After a few weeks of acclimating to the wheels, male *F/F* mice ran approximately half the daily distance of +*/*+ mice (Fig. 2f) and accordingly lost significantly less fat (Fig. 2g) while consuming the same amount of food per day (Supplementary Fig. 1K). To explore the possibility of reduced physical capability as the cause of reduced physical activity, we conducted metabolic treadmill testing in a new cohort of mice not previously exposed to running wheels. Adult *F/F* mice exhibited no deficit in either maximum rate of oxygen consumption (VO_2_max) or standardized endurance run time (Fig. 2h, i). Taken together, these results suggest that the increased adiposity of *F/F* mice is attributable to a reduced tendency for voluntary exercise.

### Paradoxically increased methylation in *F/F* ARH neurons

**Fig. 3:**
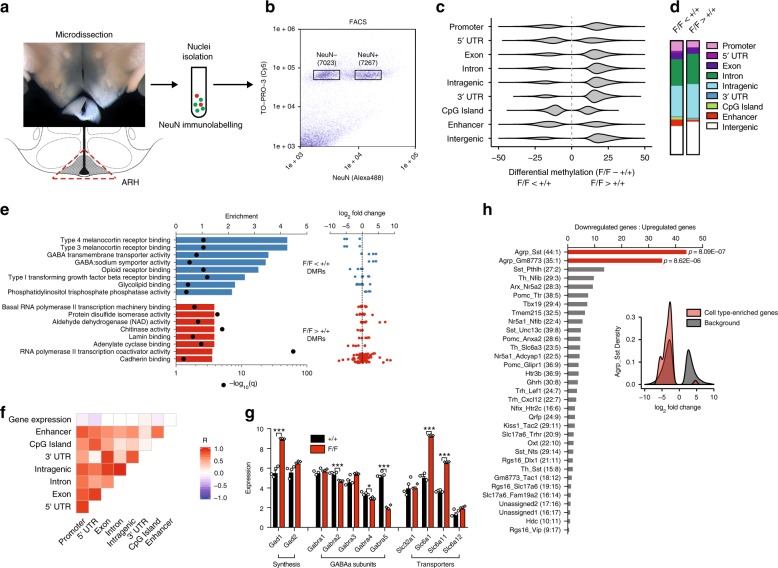
Widespread changes in DNA methylation and gene expression point to disruptions in melanocortin and GABAergic neural signaling. **a** Illustration of experimental strategy to limit cellular heterogeneity by microdissection of ARH at P12 followed by NeuN immunolabelling to isolate neuronal nuclei by FACS. **b** Representative FACS plot showing gating strategy used to isolate neural (NeuN+) nuclei (see also Supplementary Fig. 3A). **c** Violin plot showing genomic localization and methylation levels of hypomethylated and hypermethylated DMRs in ARH neurons as determined by whole-genome bisulfite sequencing. Hypermethylated DMRs predominate in *F/F* mice except in CpG island, 5′-UTR and enhancer regions. **d** Proportional genomic distribution of hypo- and hypermethylated DMRs. **e** Gene ontology (GO) function analysis of GREAT-defined^22^ *cis*-regulatory region-associated DMRs shows significant enrichment of genes associated with melanocortin signaling, GABAergic neurotransmission, and TGF-β signaling among hypomethylated DMRs (left). Genes belonging to the melanocortin signaling GO term tend to be downregulated in *F/F* mice, while genes associated with GABAergic neurotransmission tend to be upregulated in *F/F* mice (right). **f** Correlogram showing Pearson correlation coefficients (*R*) between DMR methylation level in each genomic region relative to other regions in the same gene, and expression of the associated gene. **g** Significant changes in the expression of GABA synthesis genes (*Gad1*), receptor subunits (*Gabra2, 4, 5*), and transporters (*Slc6a1, Slc6a11*). **h** Differential expression of genes specific to defined ARH neural cell types^25^. Cell types are ranked according to ratio of down- to upregulated genes characteristic of that cell type. Numbers of significantly down- and upregulated genes associated with each cell type are provided. Inset: representative density plot showing log_2_ fold change values for differentially expressed genes enriched in Agrp_Sst neurons relative to a background list of all genes with significant differential expression. *p*-Values represent results of *χ*^2^ test against background list of significantly up- and downregulated genes. Values reflect mean ± SEM; *n* = 3–5, *p < 0.05, ***p < 0.001. Source data for **g** provided as a Source Data file.

We next explored the molecular basis of this behavior change. Early postnatal life is a critical period for establishment of both de novo DNA methylation^16^ and neural projections^21^ in hypothalamic neurons. We chose to study ARH neurons from mice at P12, coinciding with the peak in *Dnmt3a* expression in the ARH (Fig. 1a) but before any body weight difference in *F/F* mice (Supplementary Fig. 1L). We FACS-purified NeuN-immunopositive nuclei from microdissected ARH samples (Fig. 3a, b, Supplementary Fig. 2A–C, Supplementary Table 1) and measured their DNA methylation by whole-genome bisulfite sequencing (WGBS) and gene expression by RNA-seq. Expression of the neuron marker *Snhg11* and the astroglia marker *Gfap* were approximately tenfold enriched and depleted, respectively, in NeuN+ vs. NeuN− fractions (Supplementary Fig. 2C) confirming effective isolation of neuronal nuclei. Our WGBS data showed that knocking out *Dnmt3a* in AgRP neurons led, paradoxically, to increased average genomic CpG methylation in ARH neurons with no change in methylation at non-CpG cytosines (i.e. CHG and CHH methylation—H corresponds to A, T, or C; Supplementary Fig. 2D). Since only CpG sites show significant differential methylation, we focused our analysis on CpG differentially methylated regions (DMRs), finding many more that were hypermethylated (i.e. *F/F* > +*/*+) (58,963) than hypomethylated (16,476) (Supplementary Fig. 2E, Supplementary Data 1). Both DMR types showed similar distributions of length and CpG density (Supplementary Fig. 2F, G). We validated several DMRs by quantitative bisulfite pyrosequencing in an independent cohort of P12 and P35 mice and found excellent agreement with our WGBS analysis (Supplementary Fig. 3A–L), both pointing to the validity of our WGBS analysis and indicating that these DMRs persist beyond weaning. Hypomethylated DMRs were relatively enriched in promoters, enhancer regions, and CpG islands (Fig. 3c, d, Supplementary Table 2). We used GREAT^22^ to associate DMRs with genes, and performed a gene ontology (GO) function analysis. Hypomethylated DMRs were associated with GO terms related to melanocortin signaling, TGF-β signaling, and GABAergic neurotransmission (Fig. 3e). Hypermethylated DMRs, on the other hand, were associated with GO terms with no obvious links to hypothalamic function, implying a less organized cellular origin (Fig. 3e). Genes in DMR-associated GO terms did not show a consistent tendency toward up- or downregulation (Fig. 3e).

### Disrupted melanocortin gene expression in *F/F* mice

Using RNA isolated from the same ARH neuronal nuclei samples that were studied by WGBS, we profiled changes in gene expression by RNA-seq. We found widespread expression changes in *F/F* mice, with 1681 up- and 2063 downregulated genes (log_2_-fold change > 1 and FDR < 0.01, Supplementary Fig. 2H, Supplementary Data 2). GO function terms related to synaptic function and neurotransmission were associated with both up- and downregulated genes (Supplementary Fig. 2I). DNA methylation in most gene-associated regions was weakly and negatively correlated with expression of the associated gene, apart from the 3′ region which showed a weak positive correlation with expression^23^ (Fig. 3f, Supplementary Fig. 2J). Surprisingly, there was negligible overlap in GO terms observed for differential methylation (Fig. 3e) and differential expression (Supplementary Fig. 2I). This is likely related to the fact that DMR methylation does not have a consistent relation to differential gene expression (Fig. 3e). Notably, GO terms related to GABA signaling were enriched in both the analysis of downregulated genes (Supplementary Fig. 2I) and hypomethylated DMRs (Fig. 3e). Specifically, *Gad1*, which encodes the primary enzyme involved in GABA synthesis, was upregulated in *F/F* neurons, whereas three of five GABAa receptor subunit genes (*Gabra2, Gabra4*, and *Gabra5*) were downregulated (Fig. 3g). To help dissociate primary, cell autonomous effects in AgRP neurons from secondary effects in other cell types, we exploited a published single-cell RNA-Seq dataset on ARH neurons^24^ to assess differential expression among genes characteristically associated with each ARH neural cell type. Strikingly, genes associated with two AgRP neuronal cell types (AgRP-Sst and AgRP-Gm8773) were almost universally downregulated (Fig. 3h). Since the number of AgRP neurons is unchanged in *F/F* mice (Fig. 1c), these data indicate downregulation of their characteristic genes, attesting to the essential role of cell autonomous DNA methylation in differentiation and cell type-specific gene expression.

### Read-level analysis identifies methylation changes in AgRP neurons

The potential for communication between neural cell types in the ARH complicates analysis of DNA methylation. While the loss of 5-mC in AgRP neurons (Fig. 1d) and coherence of our hypomethylation-associated GO terms (Fig. 3e) are consistent with our expectations, the preponderance of hypermethylation in *F/F* ARH neurons (Supplementary Fig. 2E) is more difficult to account for. One possibility is that these hypermethylated regions represent secondary effects in non-AgRP cell types. Numerous methods exist for deconvolving DNA methylation data from mixed samples^25,26^. These methods share the common goal of estimating and potentially controlling for cell type fractions in mixed samples. Our application is fundamentally different from this, as we are concerned with estimating the cellular origin of differential methylation and separating instances where increases and decreases in methylation overlap in the genome. Since each read in a WGBS library is the clonal derivative of a DNA molecule originating from a single cell, cell-specific information is inherent in WGBS data^27^, as it is in clonal BS-seq. The use of read-level bisulfite sequencing data to infer cell type- and allele-specific methylation patterns is well established in the literature^28,29^, though until now it has not yet been deployed on a genome-wide scale.

**Fig. 4:**
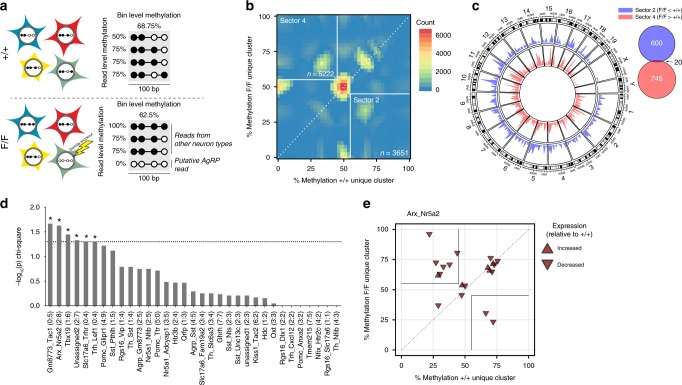
Read-level analysis of WGBS data points to epigenetic dysregulation in cell type-specific genes. **a** Illustration of strategy for pinpointing cell type-specific loss of methylation from read-level WGBS data. Searching for differentially methylated read clusters in 100 bp genomic bins enables inferences about changes taking place in individual cell types. Clusters comprise least 4 reads (see Methods); single reads are shown here for simplicity. Hypomethylated read clusters specific to *F/F* mice likely originate from AgRP neurons, whereas substantially methylated read clusters specific to *F/F* mice may arise from secondary effects in other cell types. **b** For each bin with both a cluster unique to *F/F* and a cluster unique to +*/*+ mice, average methylation levels of the unique clusters are shown in a density plot. Bins in ‘sector 2’ are defined by the simultaneous absence of a substantially methylated (≥ 55%) read cluster in +*/*+ mice and appearance of a novel hypomethylated (≤ 45%) read cluster in +*/*+ mice. ‘Sector 4’ is defined conversely. **c** Circos plot showing density of sectors 2 and 4 bins located in promoter regions of genes. Right: Venn diagram shows minimal overlap of sectors 2 and 4 promoter bins. **d** Promoter bins for differentially expressed genes specific to the molecularly defined ARH neural cell types *Gm8773/Tac1, Arx/Nr5a2, Tbx19, unassigned2, Slc17a6/Trhr*, and *Trh/Lef1*^25^ are enriched in sector 4 relative to sector 2, suggesting that these cell types contribute to the observed hypermethylation in *F/F* ARH neurons as a whole. Bars represent −log_10_(*p*) of *χ*^2^ tests comparing cell type-specific sectors 2 and 4 bins relative to a background list of all sectors 2 and 4 bins (*p < 0.05). Numbers in parentheses represent ratio of sector 2:sector 4 bin counts. **e** Plot showing the location of promoter bins for differentially expressed genes specific to *Arx/Nr5a2* ARH neurons. Plot coordinates follow the same convention as **b**.

To pursue this, we divided the genome into 100 bp bins and grouped reads with identical methylation patterns (see Methods), and used these as the basis for identifying cell type-specific differential methylation. We prototyped this approach using a published WGBS dataset from sorted neurons and glia^30^ (Supplementary Fig. 4) providing clear evidence that unique clusters of reads with identical patterns are associated with neural cell type^30^. To draw inferences specific to AgRP neurons in our mice, we sought bins in which a group of reads with substantial CpG methylation (>55%) is missing in *F/F* mice, and is replaced by a new group of reads with lower methylation (<45%) (Fig. 4a). We identified 3651 such 100 bp bins, which we refer to as ‘sector 2’ in two-dimensional Euclidian space (Fig. 4b). The opposing sector (‘sector 4’) contains 5222 bins with a unique substantially methylated cluster (and loss of a less-methylated cluster) specifically in *F/F* mice, which likely originate from non-AgRP neurons. When this procedure is carried out on randomly permuted reads, we observe substantially fewer bins in all sectors with estimated false positive rates of 4.36% and 3.2% for sectors 2 and 4, respectively (Supplementary Fig. 4D, E). Sector 2 and sector 4 bins were similarly represented in most genomic regions (Supplementary Fig. 5A) including promoters (Fig. 4c, Supplementary Fig. 5B), but sector 2 bins were enriched in CpG islands (Supplementary Fig. 5A, C; Supplementary Data 3). Although it is possible in principle for a single bin to be in both sectors 2 and 4, this occurred only 1% of the time (Fig. 4c). We found minimal overlap between hypermethylated DMRs and sector 4 bins, and similarly minimal overlap between hypomethylated DMRs and sector 2 bins (Supplementary Fig. 5D, E, respectively), indicating that this read-clustering method provides a different level of information beyond that of DMRs.

**Fig. 5:**
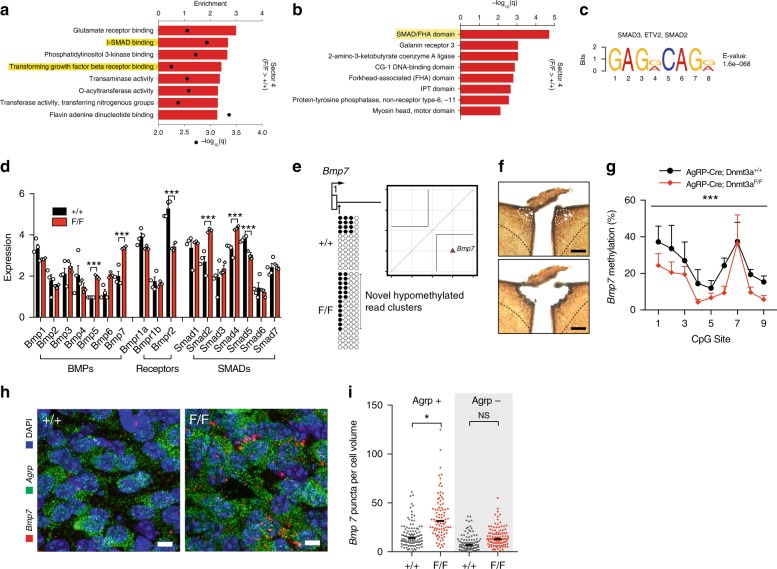
Reduced *Bmp7* methylation and increased expression in AgRP neurons lacking *Dnmt3a*. **a** Bins in sector 4 were associated with GO terms related to TGF-β and SMAD signaling. **b** Genes associated with sector 4 bins were enriched for proteins containing SMAD/FHA domains. **c** MEME-ChIP analysis shows that a SMAD2 and SMAD3 transcription factor binding motif is enriched among hypermethylated DMRs, suggesting that establishment of hypermethylation in *F/F* mice is downstream of TGF-β signaling. **d** Increased expression of bone morphogenetic protein (Bmp) signalers *Bmp5* and *Bmp7*, as well as downregulation of *Bmpr1a* and *Bmpr2* receptor subunits and altered expression of *Smad2, Smad4,* and *Smad5*. Values represent mean ± SEM. **e** Gene diagram showing location of sector 2 bin in promoter region of *Bmp7*. Tanghulu plots of read-level CpG methylation data show clusters of hypomethylated reads unique to *F/F* neurons. Inset: plot showing location of *Bmp7* promoter bin in sector 2. **f** Representative photomicrograph showing immunohistochemical localization and laser capture microdissection of AgRP neuron-enriched mediobasal ARH. Arrows indicate AgRP neurons (identified by SynTom immunostaining). White dashed lines indicate AgRP neuron-rich mediobasal ARH boundaries. Black dashed lines indicate ARH neuroanatomical boundaries. Scale bars indicate 100 μm. **g** Quantitative bisulfite pyrosequencing of the *Bmp7* promoter region identified in **j** (CpG positions 1–4) plus 100 bp downstream (CpG positions 5–9). Linear mixed model analysis indicates significantly decreased *Bmp7* methylation in the AgRP neuron-enriched mediobasal ARH (+/+ −*F/F* = −37.1% ± 4.49, df = 18.3, *p* = 0.0002). Linear mixed models were calculated using animal (*n* = 6–8) as a random factor. **h** Fluorescent in situ hybridization analysis of *Agrp* and *Bmp7* co-expression. Scale bars = 10 μm. **i** Linear mixed model analysis of fluorescent in situ hybridization data shows increased *Bmp7* expression in AgRP neurons (+/+ −*F/F* *=* −17.55 ± 6.68, df = 10.07, *p* = 0.025) but not in non-AgRP cells (+/+ −*F/F* = −6.38 ± 6.66, df = 9.89, *p* = 0.36). Linear mixed models were calculated using animal (*n* = 3) as a random factor with *n* = 90–111 cells per condition); *p < 0.05, ***p < 0.001. Source data for **d** and **g** are provided as a Source Data file.

We next asked if read-level differential methylation could be linked to a specific ARH neuronal cell type. We plotted the distribution of promoter bins for differentially expressed genes associated with each ARH neuronal cell type (Supplementary Fig. 6)^24^. Several non-AgRP neuron types show significant enrichment of differentially expressed genes with promoter bins in sector 4 (Fig. 4d), including Arx/Nr5a2 (Fig. 4e), suggesting that these cells may be the primary drivers of the hypermethylation observed in *F/F* mice. To further explore putative secondary methylation changes induced in non-AgRP neurons, we used GREAT^22^ to associate sector 4 bins with genes, finding a number of GO function terms related to TGF-β signaling and I-SMAD binding (Fig. 5a). We used InterPro to search for common protein domains among genes associated with sector 4 bins; remarkably, the SMAD/FHA domain was the top hit (Fig. 5b). In line with this, there was significant enrichment of SMAD2 and SMAD3 binding motifs among hypermethylated DMRs (Fig. 5c). Together these data indicate that SMAD binding is a common feature of regions of hypermethylation in *F/F* mice. Since the SMAD family of transcription factors are the main transducers of TGF-β signaling^31^, we next sought out potential upstream TGF-β ligands. We focused our attention on bone morphogenetic protein (Bmp) signaling^31^. Expression of both *Bmp5* and *Bmp7* was increased in *F/F* ARH neurons, while that of the Bmp receptor *Bmpr2* was decreased (Fig. 5d). Since neither *Bmp5* nor *Bmp7* are known to be expressed by ARH neural cell types^24^, we used read-level analysis of WGBS data to infer the cellular origin of this increased expression. In the case of *Bmp7*, we identified a sector 2 bin located in the promoter region of this gene (chr2:172938700–172938800) containing two unique hypomethylated read clusters in *F/F* neurons (Fig. 5e). Importantly, the *Bmp7* promoter region does not contain any DMRs, nor does average methylation differ between the genotypes (Supplementary Fig. 5F). To test whether the hypomethylated read clusters in this bin are a signature of reduced methylation of *Bmp7* specifically in AgRP neurons, we used immunohistochemical labeling of SynTom followed by laser capture microdissection (LCM) to capture the AgRP neuron-enriched mediobasal ARH (Fig. 5f). Indeed, quantitative bisulfite pyrosequencing overlapping the *Bmp7* promoter region we identified (Fig. 5e) indicated decreased methylation in *F/F* AgRP neurons relative to +*/*+ (Fig. 5g). We next used dual fluorescent in situ hybridization to localize *Bmp7* expression changes within the ARH and found increased levels of *Bmp7* expression specifically in AgRP neurons of *F/F* mice (Fig. 5h, i), consistent with the AgRP neuron-specific decreases in promoter methylation. Together, these findings support the utility of our novel read-level analysis to draw cell type-specific inferences from WGBS data, and provide mechanistic insights linking primary (cell autonomous) and secondary epigenetic alterations potentially contributing to the physical activity phenotype in this model.

## Discussion

Environmental influences during critical ontogenic periods can permanently shift an individual’s energy balance. Diverse models of such developmental programming of energy balance are characterized by alterations in energy expenditure and physical activity but not food intake^4–6^, leading us to propose that physical activity may be the most plastic or ‘programmable’ component of energy balance regulation^32^. At present the existence of a neural ‘activity-stat’^33^ regulating individual physical activity remains hypothetical and with no definitive neurobiological substrate^3^. Given the influence of central leptin signaling on physical activity^7,13,34^, such a system would likely be coupled to energy balance circuitry, and be programmable by early-life nutrition. Our discovery that DNA methylation in AgRP neurons governs voluntary physical activity meets both of those criteria, pointing to a role for epigenetic modification of hypothalamic neurons in setting the physical activity set point. This phenomenon likely depends on a range of downstream neurobiological changes, the full elaboration of which exceeds our present scope. However, several lines of evidence point to alterations in GABAergic signaling and synaptic development in ARH neurons, consistent with the established role of DNA methylation in synapse formation^35^ and the primarily GABAergic nature of AgRP neurons^36^. Given that AgRP neurons are GABAergic^36^, simultaneous disruption of GABA synthetic enzyme and receptor subunit expression could point toward broader, network-level changes in neural signaling, potentially brought about by disrupted differentiation of AgRP neurons in *F/F* mice.

Our use of AgRP-IRES-Cre mice means that the onset of the *Dnmt3a* knockout coincides with the onset of *Agrp* expression. In mice, this takes place in the late embryonic and early postnatal period^37^, during which hypothalamic neurons gain significant amounts of DNA methylation^16^. DNA methylation in the brain regulates gene expression, but is itself established secondary to gene expression during development^38,39^. This, and the fact that epigenetic modifications affect gene expression *potential*, is consistent with our observations of an imperfect correspondence between differential methylation and expression, though the two were broadly correlated. These points do leave open numerous questions regarding the exact timing of de novo DNA methylation and de-methylation and their role in dynamic epigenetic and gene expression changes in mature cells^40,41^. Future studies using inducible *Dnmt3a* knockdown/reinstatement would be ideally suited for such questions.

Physical activity is an important component of the energy balance equation, though it has a complicated relationship with energy expenditure and body composition. Since neither wheel running nor home cage locomotion are resistance exercises, they do not increase muscle mass^42^. Running wheel access does reliably increase rates of physical activity and total energy expenditure, though its effects on body weight and composition depends in large part on potential compensatory increases in food intake^43^. The fundamental cause of the reduced exercise in *F*/*F* mice remains mysterious. Although our metabolic treadmill results suggest a volitional origin, further study using larger samples sizes and complementary measures of volition will be required to confirm this.

Having perturbed a fundamental mechanism of transcriptional regulation in AgRP neurons, a major challenge lies in determining relevant downstream mediators of the observed phenotype. This imperative exists for every study of cell type-specific genetic disruption, because the resulting phenotype depends not only on the loss of function in one cell type, but also on how other cells in the system respond. Compensatory effects abound in the energy balance circuitry; indeed, mice can survive early-life ablation of AgRP neurons^44^ with only a subtle adult phenotype^45^. Similar compensatory mechanisms may underlie the contrast between the numerous transcriptional and epigenomic changes we found at P12 and the comparatively mild adult adiposity phenotype we observed.

To begin to understand downstream, cell type-specific effects, we implemented a read-level analysis of our WGBS data, analogous to previously described approaches using ultra-deep BS-seq to identify minor cell populations in pituitary tissue^29^. This led us to conclude that TGF-β-associated SMAD binding motifs enriched in hypermethylated DMRs are a signature of secondary effects in non-AgRP cell types, and that specific hypomethylated read clusters in the promoter region of *Bmp7* originate from AgRP neurons. *Bmp7*, a TGF-β ligand^31^ implicated in hypothalamic development^46,47^, is upregulated specifically in AgRP neurons of *F/F* mice. *Bmp7* influences axon guidance in hypothalamic neuroendocrine cells^48^, and deletion of *Bmpr1a* in *Olig1* hypothalamic progenitors significantly decreases AgRP and POMC neuronal projections to the PVH^49^. Overexpression of *Bmp7* (caused by AgRP neuron-specific promoter hypomethylation) may thus explain both cell autonomous developmental effects within AgRP neurons and, by virtue of being a secreted factor, effects on methylation in neighboring neurons. Still, *Bmp7* was one of thousands of differentially expressed genes and while numerous lines of evidence suggest its involvement, it is unlikely a solitary actor—which will complicate the interpretation of potential directed overexpression follow-up experiments. While further studies will be needed to fully investigate the role of Bmp7 and potential other players, and to conclusively link developmental epigenetics in the melanocortin system to adult physical activity, our work provides a novel insight that neuroepigenetic development is a key determinant of voluntary exercise behavior.

## Methods

### Animals

This study was approved by the Institutional Animal Care and Use Committee of Baylor College of Medicine, Childrens Hospital of Los Angeles, and Vanderbilt University, and animals were maintained in accordance with federal guidelines. Mice were housed in same-sex groups of 2–4 for most experiments, and maintained on a 12-h light/dark cycle (lights on at 6:00 AM) at 22 °C with ad libitum access to food and water in glass bottles. NPY-hrGFP^50^ mice (Jackson Laboratories, RRID:IMSR_JAX:006417) express humanized *Renilla* GFP under the control of the NPY promoter, and because NPY is co-expressed with AgRP in the ARH, these mice were used for initial characterization of co-expression of DNMT3A and NPY. Pregnant and lactating dams were fed a fixed-formula, soy protein-free reproductive diet (2919X; Envigo), while weanlings and adults were fed a fixed-formula, soy protein-free maintenance diet (2020X; Envigo). *AgRP-IRES-Cre*^*+*^; *Dnmt3a*^*F/F*^ (F/F) mice were generated by crossing *AgRP-IRES-Cre* mice^51^ (Agrptm1(cre)Lowl, Jackson Laboratories, RRID:IMSR_JAX:012899) with mice possessing loxP sites flanking exon 18 of the *Dnmt3a* gene^52^. *AgRP-IRES-Cre*^*+*^; *Dnmt3a*^*F/F*^*; SynTom*^*+*^ mice were generated by crossing *AgRP-IRES-Cre*^*+*^; *Dnmt3a*^*F/F*^ mice with mice harboring a Synaptophysin-TdTomato fusion protein knocked into the Gt(ROSA)26Sor locus under the control of a loxP-flanked STOP cassette (Ai34(RCL-Syp/tdT)-D, Jackson Labs, RRID:IMSR_JAX:012570). All experiments were performed using Cre-expressing mice, with animals possessing wild-type Dnmt3a alleles (+/+ mice) as controls in order to control for possible metabolic effects of Cre expression^53^. Except where noted, all experiments were performed in male mice.

### Metabolic phenotyping

Body weight was measured with a calibrated integrating scale and body composition by quantitative magnetic resonance (QMR) (EchoMRI-100; EchoMedical Systems LLC, Houston, TX, USA), according to the manufacturer’s instructions. Metabolic phenotyping was carried out in male and female mice at 20 weeks of age. Prior to beginning metabolic measurements, mice were individually housed for 3 days in feeder cages, to acclimate them to the conditions of metabolic analysis. Mice were then individually placed into Comprehensive Laboratory Animal Monitoring System (CLAMS) cages (Columbus Instruments)^5^. Briefly, measures of VO_2_, VCO_2_, food intake, and locomotor activity were collected at half-hour intervals for three consecutive days. Energy expenditure and X-axis ambulatory locomotion were calculated by the included Oxymax software package (Columbus Instruments) from raw VO_2_, VCO_2_, RER, and X-axis beam break data. Data from the first 24 h were discarded to account for acclimation to the metabolic cages. Analysis and figures are based on average values obtained over the two remaining days of recording. Estimates of non-activity-related energy expenditure were carried out beginning at 12:00 PM (ZT6) on the fourth day. During this period, mice are naturally physically inactive and consume minimal food. To further minimize the influence of the thermic effect of food, access to feeders was blocked beginning at 6:00 AM. Two energy expenditure measures coincident with the two least physically active time bins were averaged to estimate non-activity related energy expenditure in each mouse. Energy expenditure and food intake measures were normalized to lean and fat mass using ANCOVA. Metabolic data, including energy expenditure and food intake, are presented as least-squares means derived from lean and fat mass-adjusted ANCOVA.

### Running wheels

Running wheel trials were carried out in two independent cohorts of mice. At 32 weeks of age, mice were singly housed in rat cages equipped with solid surface, 14 cm diameter running wheels (Kaytee) modified to run on ball bearings. Rotations were counted in 10 min timebins using reed switches connected to Arduino microcontrollers with Adafruit data logging shields (Adafruit). At 0, 2, 4, 6, and 8 weeks of wheel exposure, body composition was measured by QMR. This analysis was conducted in two separate cohorts of mice. Weekly average food intake was collected from mice in the first cohort.

### Metabolic treadmill

Treadmill testing was performed on adult mice as described previously^54^. All testing took place between 10 AM and 12 PM. Mice were placed in individual lanes of an Exer3/6 mouse/rat treadmill (Columbus Instruments) and subjected to a graded habituation protocol. On the first day, mice were left undisturbed in the treadmill apparatus for 15 min. On the following 4 days, mice were subjected to a graded increase in speed (5–10 m/min), grade (0–10°) and electric shock intensity (0–50%), until they were ultimately running at 10 m/min on a 10° incline under threat of a 50% electrical shock.

After at least 2 days of rest, mice were placed in Metabolic Modular Treadmills (Columbus Instruments) connected to the CLAMS system. The test began with 5 min of habituation (belt off) followed by 8 min at 10 m/min with no incline. After this, the incline was increased to 10° and 4 min later the speed was increased by 2 m/min. Subsequently, the speed was increased an additional 2 m/min every 2 min. During this time VO_2_, VCO_2_, and RER were measured. The experimental end point was defined when RER reached or exceeded 1.0, or if the mouse was unable to escape the shock grid (more than five shocks in a 30-s period). VO_2_max was determined when oxygen consumption failed to increase despite increasing treadmill speed.

After at least 4 days of rest, mice were returned to the Exer3/6 treadmill. The endurance exercise test began with an initial warm up at 10 m/min with a 0° incline for 8 min. The incline was then raised to 10°. After 2 min, the speed was raised by 5 m/min until the speed that is 60% of the maximum workload achieved on the stress test is attained (between 20 and 25 m/min). Mice were allowed to run until exhaustion. Exhaustion was defined as either remaining on the shock grid for 5 s, or falling off the back of the belt five times in 30 s. Endurance was defined as the number of minutes mice ran before reaching exhaustion.

### Immunofluorescence histology

P10 NPY-hrGFP mice were deeply anesthetized and sacrificed by perfusion with 0.9% saline, followed by 2% paraformaldehyde. Brains were dissected free and fixed overnight in the same fixative at 4 °C. P35 +*/*+ and *F/F* immersion fixed brains were used for analysis of 5-mC and 5-hmC labeling. Brains were cryoprotected in 30% sucrose. Cryosections of P10 brains were collected onto glass slides and stored at −80 °C prior to staining. P35 brains were cryosectioned at 40 μm in a 1-in-4 series. The following antibodies were used for immunofluorescence: RFP (Abcam ab34771, RRID:AB_777699, 1:1000), DNMT3A (Abcam ab2850, RRID:AB_303355, 1:1000, 1:1000), 5-mC (Cell Signaling Technology 28692, 1:100), 5-hmC (Abcam ab214728, 1:100). All washing steps were performed using three changes of 1× PBS. All antibody incubations took place in blocking buffer (1% BSA, 0.3% Triton-X 1000 in PBS). After secondary antibody incubation, sections were washed with PBS and mounted on glass slides. After drying briefly, slides were coverslipped using ProLong Diamond Antifade Mountant with DAPI (Thermo Fisher). No-primary antibody controls were run during initial antibody titration. *DNMT3A:* after washing and incubating in blocking solution, slices were incubated for 48 h in rabbit anti-DNMT3A (Abcam ab2850, RRID:AB_303355, 1:1000) at 4 °C. Slices were developed in Alexa594-conjugated donkey anti-rabbit IgG (Thermo Fisher R37119; RRID:AB_2556547, 1:500) for 1 h at room temperature. *5-methylcytosine:* in order to render DNA accessible to antibody detection, samples were incubated in 1 M HCl for 30 min at 45 °C. Sections were washed in PBS, then incubated in 0.3% glycine for 10 min at room temperature. Sections were washed, then incubated for 1 h in blocking buffer. Sections were incubated overnight at room temperature in blocking buffer containing the rabbit anti-5-mC (Cell Signaling Technology 28692, 1:100) primary antibody. The next day, sections were washed and developed using Alexa488-conjugated donkey anti-rabbit IgG (Thermo Fisher A-21206, 1:500). After washing, sections were incubated overnight in biotinylated anti-RFP (Abcam ab34771, 1:1000) at room temperature. The next day, sections were developed using Alexa647-streptavidin conjugate (Thermo Fisher S32357, 1:500) for 1 h at room temperature. *5-hydroxymethylcytosine:* 5-hmC staining was carried out similarly to 5-mC staining, with the exception of an antigen retrieval step (10 mM Sodium citrate, 0.05% Tween 20, pH 6.0 for 20 min at 95 °C) prior to acid treatment. Primary antibody incubation in rabbit anti-5-hmC (Abcam ab214728, 1:2000) took place overnight at room temperature. This combination of steps is needed to enable 5-hmC staining, but also renders RFP/SynTom labeling ineffective.

### Confocal microscopy and image analysis

Immunofluorescence images were captured on a Leica DMi8 laser scanning confocal microscope. For 5-mC and 5-hmC staining, *Z*-stacks were captured at 0.5 μm intervals through at least three ARH sections (−1.46 mm to −2.06 mm relative to Bregma) per animal at 10× magnification. 5-mC was quantified by mean fluorescence intensity in AgRP neurons based on co-localization with SynTom-labeled cell bodies. To estimate whether loss of *Dnmt3a* lead to loss of AgRP neurons, we also compared the number of SynTom-labeled cell bodies in anatomically matched sections between the two genotypes. Because the antigen retrieval process for 5-hmC rendered RFP/SynTom labeling ineffective, we analyzed the intensity of 5-hmC labeling in neurons located in the mediobasal region of the ARH, which contains the highest density of AgRP neurons. Mean fluorescence intensity of these cells was compared to that of cells located in the dorsolateral ARH, a region that lacks AgRP neurons, to obtain a rough estimate of altered 5-hydroxymethylation in AgRP neurons.

### Tissue preparation

Brains from +*/*+ and *F/F* mice (*n* = 5 per genotype) were collected at P12 following sacrifice by decapitation. Brains were removed and placed into ice-cold PBS then flash-frozen whole on powdered dry ice. ARH microdissections including the median eminence were performed on two consecutive 200-μm coronal vibratome sections from each brain under a dissecting microscope. Triangular-shaped cuts were made along the morphological boundaries of the nucleus (Fig. 3a). ARH microdissections were flash frozen on dry ice and stored at −80 °C. ARH microdissections were dissociated by Dounce homogenization, and nuclei were purified by ultracentrifugation against a 1.8 M sucrose column at 100,000 rcf. Purified nuclei were stained using rabbit anti-NeuN (Millipore ABN78; 1:4000) followed by Alexa488-conjugated goat anti-rabbit IgG (Thermo Fisher A-11008; 1:2000) and the nucleic acid dye TO-PRO-3 as a counterstain (Thermo Fisher; 1:1000). Sorting was performed on a Sony SH800 Cell Sorter (Sony Biotechnology). FSC and SSC gates were established to eliminate debris and non-nuclear material (Supplementary Fig. 3A), and TO-PRO-3 gating was used to collect single nuclei only. NeuN− and NeuN+ populations were sorted on the basis of antibody staining in the sorter’s Purity mode^16^. Nuclei were immediately frozen on dry ice and stored at −80 °C. ARH NeuN+ nuclei were homogenized in buffer RLT and DNA was extracted using the AllPrep DNA/RNA Micro Kit (QIAGEN) according to the manufacturer’s instructions. DNA was eluted from columns using two rounds of 50 μl nuclease-free H_2_O, pH 8.0, dried in a SpeedVac (Eppendorf), and resuspended in 12 μl of TE buffer (pH 8.0). DNA was quantitated using the PicoGreen assay (Thermo Fisher) as directed by the manufacturer. RNA was prepared from flow-through using 1000 μl of RNA STAT-60 (Tel-Test Inc.) according to the manufacturer’s instructions and resuspended in 12 μl of nuclease-free H_2_O. RNA was quantitated by NanoDrop spectrophotometry (Thermo Fisher).

### qPCR

RNA was extracted from ARH microdissections from neonatal C57BL/6 mice at P2, P10, P12, and P21 using RNA STAT-60 (Tel-Test Inc.) according to the manufacturer’s instructions and resuspended in 12 μl of nuclease-free H_2_O. RNA was quantitated by NanoDrop spectrophotometry (Thermo Fisher). First-strand cDNA synthesis was performed using M-MLV reverse transcriptase (Promega) and random oligo primers (Thermo Fisher) according to the manufacturer's directions. qPCR for *β-actin* and *Dnmt3a* was performed using TaqMan assays (Mm02619580_g1 and Mm00432881_m1, respectively) according to the manufacturer’s instructions.

### Bisulfite sequencing

Tagmentation-based whole-genome bisulfite library preparation was performed on genomic DNA extracted from ARH NeuN+ nuclei^55^. Adaptors were pre-annealed and transposomes were assembled according to the protocol described in Wang et al.^55^; 10–30 ng of mouse genomic DNA was tagmented by adding the assembled transposome and incubating at 55 °C for 8 min. Following purification using SPRI beads, oligonucleotide replacement and gap repair were performed using Ampligase and T4 DNA Polymerase. The product was purified using SRPI beads and followed by bisulfite treatment using the EZ DNA Methylation Gold kit (Zymo Research). Subsequently libraries were generated by PCR amplification using KAPA 2G robust hotstart ready mix. After purification using SPRI beads, libraries were diluted and sequenced on an Illumina Hiseq2500 Sequencer in 150 bp paired-end mode.

### RNA sequencing

Libraries for RNA sequencing were prepared using the SMART-Seq v4 Ultra Low Input kit (Takara Clontech) according to the manufacturer’s instructions. Briefly, purified RNA was incubated with lysis buffer for 5 min. First-strand cDNA synthesis was performed using the included 3-SMART-seq CDS primer II and V4 oligonucleotide. cDNA was amplified using PCR Primer II A, and subsequently purified using Ampure XP beads (Beckman). Illumina libraries were prepared using Nextera XT DNA library preparation kit (Illumina), and sequenced on the Illumina Hiseq 2500 platform generating 100 bp paired-end reads.

### WGBS analysis

Fastq reads were quality trimmed using TrimGalore (v0.4.3) with a minimum quality score of 20 and a minimum post-trimming read length of 50. Read quality was analyzed before and after trimming using Fastqc (v0.11.5). After trimming, the reads were aligned to the mouse genome (mm10) using Bismark (v0.18.1) set to paired-end mode and using all other default settings. Read groups corresponding to each individual sample were added to these BAM alignments using Picard (v2.10.10). ‘AddOrReplaceReadGroups’ BAM files were query-name sorted using Picard ‘SortSam’, and then deduplicated using Picard ‘MarkDuplicates’. One library was removed from the analysis due to high PCR duplicate rate (85%) and low coverage (1.2× average coverage). Coverage for the remaining libraries totaled 45× for +*/*+ and 47× for *F/F*. Differentially methylated CpG loci (DMLs) were identified by DSS^56^ using the general experimental design with an FDR cutoff of 0.05. Only CpGs with at least five reads per library were considered. DMRs were subsequently identified by DSS with a *p*-value threshold of 0.05 and an absolute delta cutoff of 10% between the genotypes. DMRs were annotated to UCSC gene features, with promoter regions defined as ±2.5 kb from the canonical transcription start site, enhancers were identified by a combination of DNAse sensitivity and H3K27ac enrichment (ENCODE Encyclopedia, Version 3 http://zlab-annotations.umassmed.edu/enhancers/). Intergenic DMRs were defined as any DMR not overlapping a gene body, promoter, or 3′ region. Hypo- and hypermethylated DMRs were separately associated with *cis*-regulatory regions by GREAT v3.09, and the top eight enriched GO process terms for each were reported^22^. Hypermethylated DMRs were analyzed by MEME-ChIP using the default settings^57^.

### RNA-seq analysis

Fastq reads were quality trimmed using TrimGalore (v0.4.4) with a minimum quality score of 20 and a minimum read length of 50. Read quality was analyzed using fastqc (v0.11.5). Quality-trimmed reads were aligned to the mouse genome (mm10) using Hisat2 (v2.1.0). Mapped reads were annotated and counted using featureCounts (v1.5.3) along with the gencode comprehensive gene annotation (gencode vM15 gff3 file). Differential expression testing was performed using DESeq2 (v1.16.1). Genes showing significant (FDR *q* value <0.01, absolute log_2_ fold change ≥1) between genotypes were used for GO analysis in separate lists (www.geneontology.org). The top eight GO process terms (sorted by enrichment, FDR < 0.001) for both up- and downregulated genes were reported. Expression and gene-associated DNA methylation were correlated using Pearson’s product moment correlation coefficient with values collapsed over genotype. To plot our gene expression results against ARH cell type-specific enrichment, we first selected the top 100 genes for enrichment in each of the neural cell types defined by Campbell et al.^24^. For each of these lists, we plotted the ratio of highly (FDR *q* value <0.01, absolute log_2_ fold change ≥2) up- and downregulated genes in our RNA-Seq results. We used *χ*^2^ to analyze these ratios, comparing the number of up- and downregulated genes in each cell type class to the overall number of significantly up- and downregulated genes detected. *p*-Values were adjusted for multiple testing by Bonferroni correction.

### Read-level WGBS analysis

The mouse genome was divided into non-overlapping 100 bp windows. Mapped BAM files were query- name sorted and deduplicated using Picard (v2.10.10) and coordinate sorted using SAMtools (v1.9). For each 100 bp window, all mapped reads were extracted from coordinate-sorted BAM files. The methylation state of each CpG site was extracted for every read. To ensure paired-end reads originating from the same DNA fragment were not counted twice, we discarded overlapping bases originating from the same read pair. When possible, the remaining bases on the trimmed read were stitched onto the first read, maximizing the amount of information obtainable. We excluded any reads not fully covering all CpGs within the 100 bp window, and the remaining read data were used to create a *m* × *n* read-methylation matrix, with *m* representing the number of reads and *n* representing the number of CpG sites. The matrix was populated with values of 0 and 1 representing unmethylated and methylated CpGs, respectively.

A read-methylation matrix was generated for both +*/*+ and *F/F* samples. If both matrices had at least 10 reads, the matrices were combined. Reads containing identical methylation patterns were grouped together using the DBSCAN method from the scikit-learn package^58^. Any group with <4 reads was dropped. The methylation pattern, methylation level, number of reads, and genomic location of each group was reported from the remaining groups. This method was carried out for every 100 bp window in the genome.

To estimate the rate of spurious read cluster detection, we performed a permutation test. For each 100 bp bin, the genotype identification of each read was randomly re-assigned. Re-assigned reads were labeled either A or B, and read clustering was then performed as described above. This process was repeated over 10 iterations. False-positive rates were calculated as the average number of bins detected in each sector of permuted data divided by the number detected in the unpermuted data.

### Bisulfite pyrosequencing

A subset of DMRs were validated in a separate cohort of ARH neurons from mice sacrificed at P12 and P35 by bisulfite pyrosequencing using a Biotage Pyromark MD pyrosequencing system^16^. Prior to use in validation, pyrosequencing assays were assessed for sensitivity and linearity by running standard curves consisting of known mixtures of fully methylated and unmethylated mouse DNA (0, 25, 50, 75, and 100% methylation)^59^. Assay primers are provided in Supplementary Table 3.

### ISH analysis

Two-color fluorescent in-situ hybridization was performed on 15 μm fixed frozen slices from P12 male +*/*+ and *F/F* mice mounted on Superfrost Plus slides (Fisher Scientific) using probes and directions provided by Advanced Cell Diagnostics (ACD Technical notes #320535 for tissue prep, and #320295 for Fluorescent Multiplex kit, http://www.acdbio.com/technical-support/downloads). A channel 2 probe against Agrp was used to identify AgRP neurons (Mm-Agrp-C2, 400711-C2). Bmp7 (Mm-Bmp7, 407091) was used as a target probe. Slides were counter-stained using DAPI and coverslipped with ProLong Diamond Antifade Mountant (ThermoFisher). Sections were imaged on a Leica DMi8 laser scanning confocal microscope at 60×. *Z*-stacks at 0.5 μm increments were captured through the ARH in anatomically matched sections. Quantification of mRNA was performed as previously described^60^, using cell volumes from maximum intensity projections of acquired *Z*-stacks using the StarSearch Java applet with identical threshold settings for each image (http://rajlab.seas.upenn.edu/StarSearch/ launch.html).

### Laser capture microdissection

LCM of AgRP neuron-enriched ARH samples was performed using fixed frozen 40-μm coronal tissue sections from male and female P12 +*/*+ and *F/F* SynTom+ mice^61^. Sections were incubated overnight at room temperature in blocking buffer containing the rabbit anti-RFP (Abcam ab623421, 1:1000) primary antibody. Following this, sections were washed and developed using the rabbit-specific HRP/DAB detection kit (Abcam ab64261) according to the manufacturer’s directions. Sections were mounted on PEN membrane slides (ThermoFisher) prior to LCM. LCM was performed on an ArcturusXT LCM system (ThermoFisher) using the dual laser mode to microdissect and retrieve the AgRP neuron-enriched mediobasal ARH with immunohistochemical guidance. Microdissections were collected onto CapSure Macro LCM caps (Thermo Fisher) and DNA was extracted, bisulfite converted, and pyrosequenced as described above.

### Statistics

SPSS 17 (IBM) and R 3.4 (R Foundation for Statistical Computing) were used for all statistical analysis. To minimize the influence of litter effects in these studies, mice from at least three separate litters were used for all experiments. Except where noted, the experimental unit of analysis was individual mice. Genotype effects were compared using two-tailed independent samples *t*-tests. Time-course measures such as body weight and body composition were analyzed using repeated measures ANOVA with genotype and sex as the between-subjects factors. Calorimetry data were analyzed using repeated measures ANCOVA, with lean and fat body mass as the covariate. Data from these experiments are presented as the least-squares means from the model including lean and fat body mass. Locomotor data, including beam break measures taken in the calorimetry system, were analyzed using repeated measures ANOVA. Analysis of bisulfite pyrosequencing and in situ hybridization was performed using a linear mixed model approach with the R package *lme4*, with genotype, age, sex, CpG position, and cell type serving as fixed factors where appropriate, and animal serving as a random factor. Post-hoc analysis of fixed effects was performed using linear contrasts. Statistical analysis of and RNA-Seq and WGBS data are explained in their respective sections.

### Reporting summary

Further information on research design is available in the Nature Research Reporting Summary linked to this article.

## Supplementary information

Supplementary Information

Description of Additional Supplementary Files

Supplementary Data 1

Supplementary Data 2

Supplementary Data 3

Reporting Summary

## Data Availability

All described sequencing data (WGBS and RNA-Seq) have been deposited in the Gene Expression Omnibus (GEO) (Accession number: GSE122405). Processed data including DMR locations, RNA-Seq expression values, and regions identified by read-level analysis are available in Supplementary Data 1, 2, and 3, respectively. The source data underlying Figs. 1a, c, d, 2a–i, 3g, 5d, g, Supplementary Figs. 1D–L, 2C, and 3A–L are provided as a Source Data file.

## Source data

Source Data
