# Supplementary material for: DNA methylation in AgRP neurons regulates voluntary exercise behavior in mice
Source: Nat Commun. 2019 Dec 2;10:5364. doi: 10.1038/s41467-019-13339-3 (PMC6889160; doi:10.1038/s41467-019-13339-3)
Supplement: Supplementary file 1 — Supplementary Information [file 41467_2019_13339_MOESM1_ESM.pdf]

Supplementary Information

DNA methylation in AgRP neurons regulates voluntary exercise behavior

MacKay et al.,

Table of contents:

Supplementary figures:

1. Additional mouse phenotype data.
2. Additional details on FACS, DMR and RNA-seq data.
3. Results of bisulfite pyrosequencing DMR validation.
4. Demonstration of read-level analysis on sorted neuron and glia WGBS data; results of permutation testing.
5. Additional information on read-level WGBS analysis.
6. Promoter-associated bins of interested from read-level WGBS analysis in cell type-enriched gene sets.

Supplementary tables:

1. Description of samples used and excluded in RNA-seq and WGBS experiments.
2. Genomic location, count, and length statistics for DMRs (F/F-+/+).
3. PCR and sequencing primers for bisulfite pyrosequencing assays.

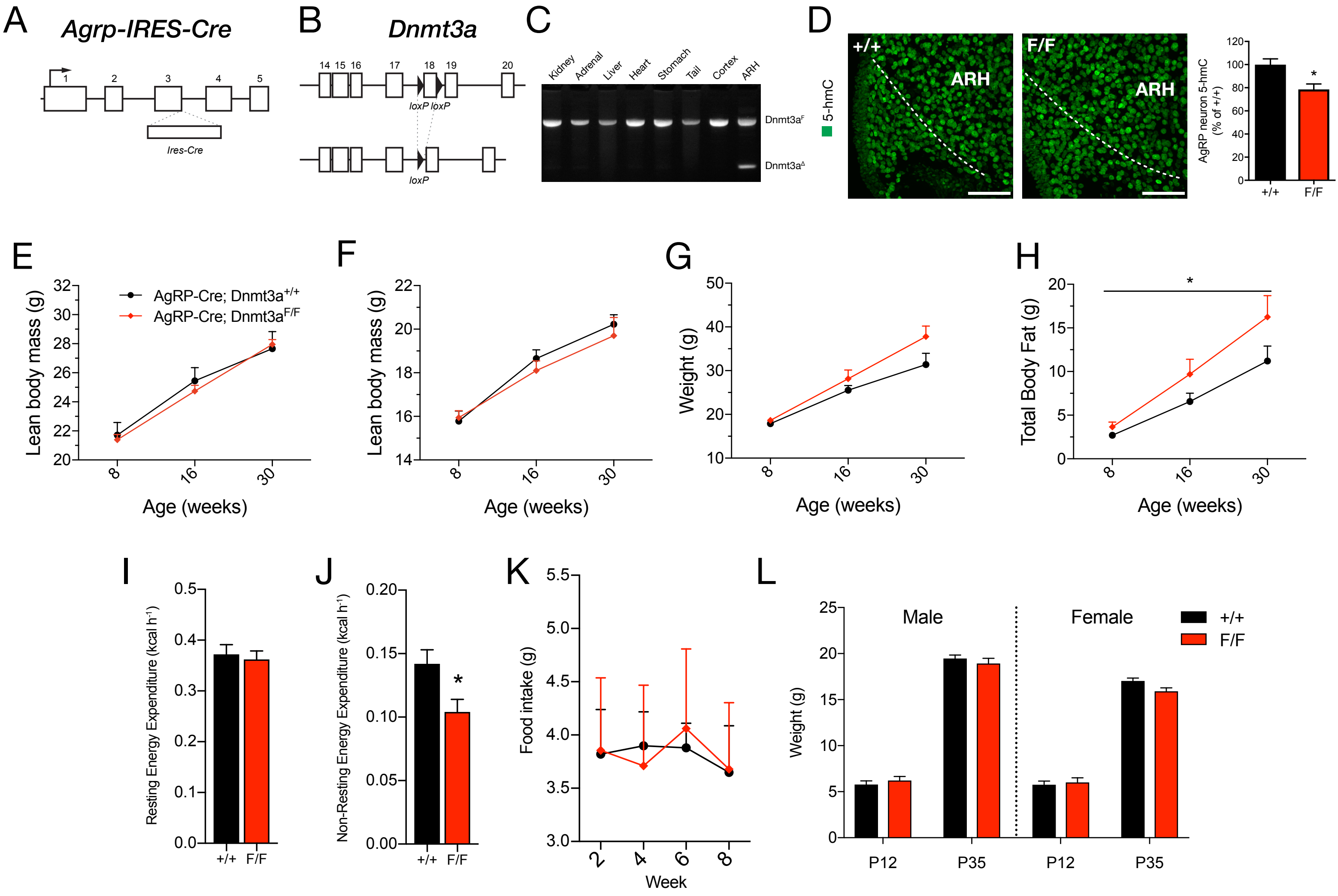

Supplementary Figure 1

(A) Schematic of Agrp-IRES-Cre locus. (B) Schematic of floxed Dnmt3a allele – above: 2 loxP intact allele, below: 1 loxP knockout allele. (C) PCR validation using primer sets specific to 2lP and 1lP Dnmt3a alleles, showing ARH-specific Dnmt3a recombination in F/F mice. (D) Putative AgRP neurons (identified by mediobasal ARH location) show reduced 5-hydroxymethyl cytosine levels relative to non-mediobasal ARH cells in F/F mice $t(5)=3.09$, $p=0.0271$ (two-tailed). Scale bars = 10 µm. (E) No difference in lean body mass in male F/F mice. (F) No difference in lean body mass in female F/F mice. (G) Female F/F mice show no difference in body weight relative to +/+ mice, $F(1,32)=3.56$ main effect of Genotype, $p=0.068$, (n=6-12). (H) Increased adiposity in female F/F mice, $F(1,33)=4.75$ main effect of Genotype, $p=0.036$, (n=6-13). (I) Resting metabolic rate does not differ between male +/+ and F/F mice, $F(1,8)=0.141$, $p=0.717$ main effect of genotype (ANCOVA with lean and fat body mass as covariates). (J) Non-activity associated energy expenditure is significantly reduced in male F/F mice, $F(1,8)=5.635$, $p=0.045$ main effect of genotype (ANCOVA with lean and fat body mass as covariates). (K) No effect of genotype on lean and fat body mass-adjusted food intake in mice given access to running wheels (n=7-8). Energy expenditure and food intake data are presented as least-squares means, adjusting for lean mass and fat mass. (L) No significant genotype effects on body weight in either sex at P12 or P35 (n=4-12). Values represent mean +/- SEM. * $p < 0.05$, *** $p < 0.001$

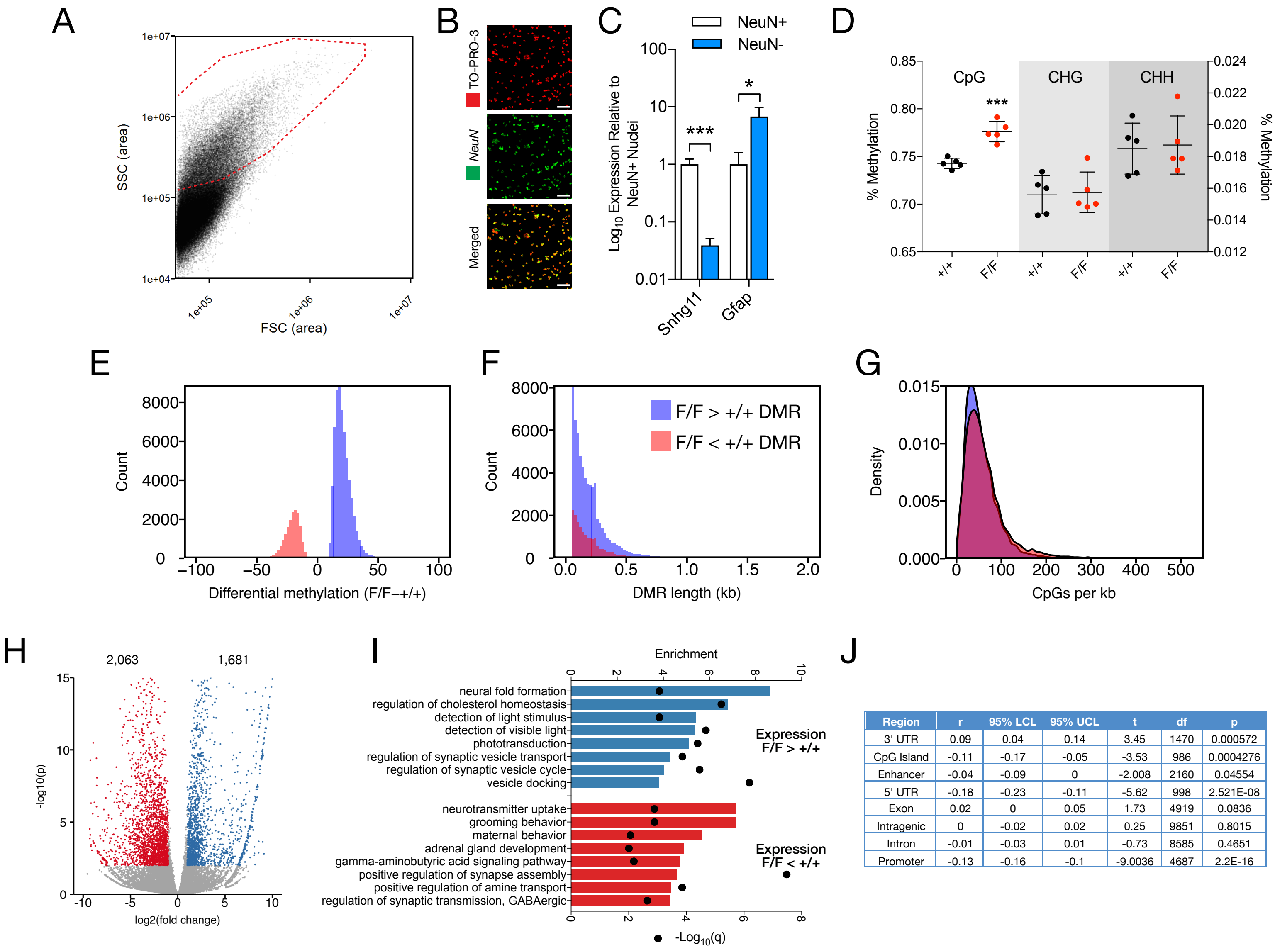

Region	r	95% LCL	95% UCL	t	df	p
3' UTR	0.09	0.04	0.14	3.45	1470	0.000572
CpG Island	-0.11	-0.17	-0.05	-3.53	986	0.0004276
Enhancer	-0.04	-0.09	0	-2.008	2160	0.04554
5' UTR	-0.18	-0.23	-0.11	-5.62	998	2.521E-08
Exon	0.02	0	0.05	1.73	4919	0.0836
Intragenic	0	-0.02	0.02	0.25	9851	0.8015
Intron	-0.01	-0.03	0.01	-0.73	8585	0.4651
Promoter	-0.13	-0.16	-0.1	-9.0036	4687	2.2E-16

Supplementary Figure 2

(A) Representative FACS plot showing strategy for gating intact nuclei. (B) Representative photomicrograph showing NeuN labeling in nuclei isolated from ARH microdissections. Scale bars = 50 µm. (C) Validation of FACS sorting by qPCR evaluation of neuron-specific (Snhg11; t(6)=3.93, p=0.0038 two-tailed) and astrocyte-specific (Gfap; t(6)=1.95, p=0.049 two-tailed) in NeuN+ and NeuN- populations (n=4). (D) F/F mice have significantly higher levels of methylated CpGs (t(8)=6.22, p=0.0003 two-tailed), but no differences in CHG or CHH methylation. (E) Histogram showing differential methylation (F/F - +/+) values for all DMRs. (F) Hypo- and hyper-methylated DMRs do not show any difference in length. (G) CpG density does not differ between hypo- and hyper-methylated DMRs. (H) Volcano plot illustrating genes showing increased and decreased expression in F/F mice. (I) GO function analysis of genes showing significant differential expression in RNA-Seq experiment (log2-fold change > 1, FDR<0.01) (n=3-4). (J) Table showing Pearson correlation coefficients (r), upper (UCL) and lower (UCL) confidence limits, t-test value, degrees of freedom (df) and p value for relationship between DMR methylation level and expression of the associated gene for each genomic annotation. Summary statistics represent mean +/- SEM. * $p < 0.05$, *** $p < 0.001$

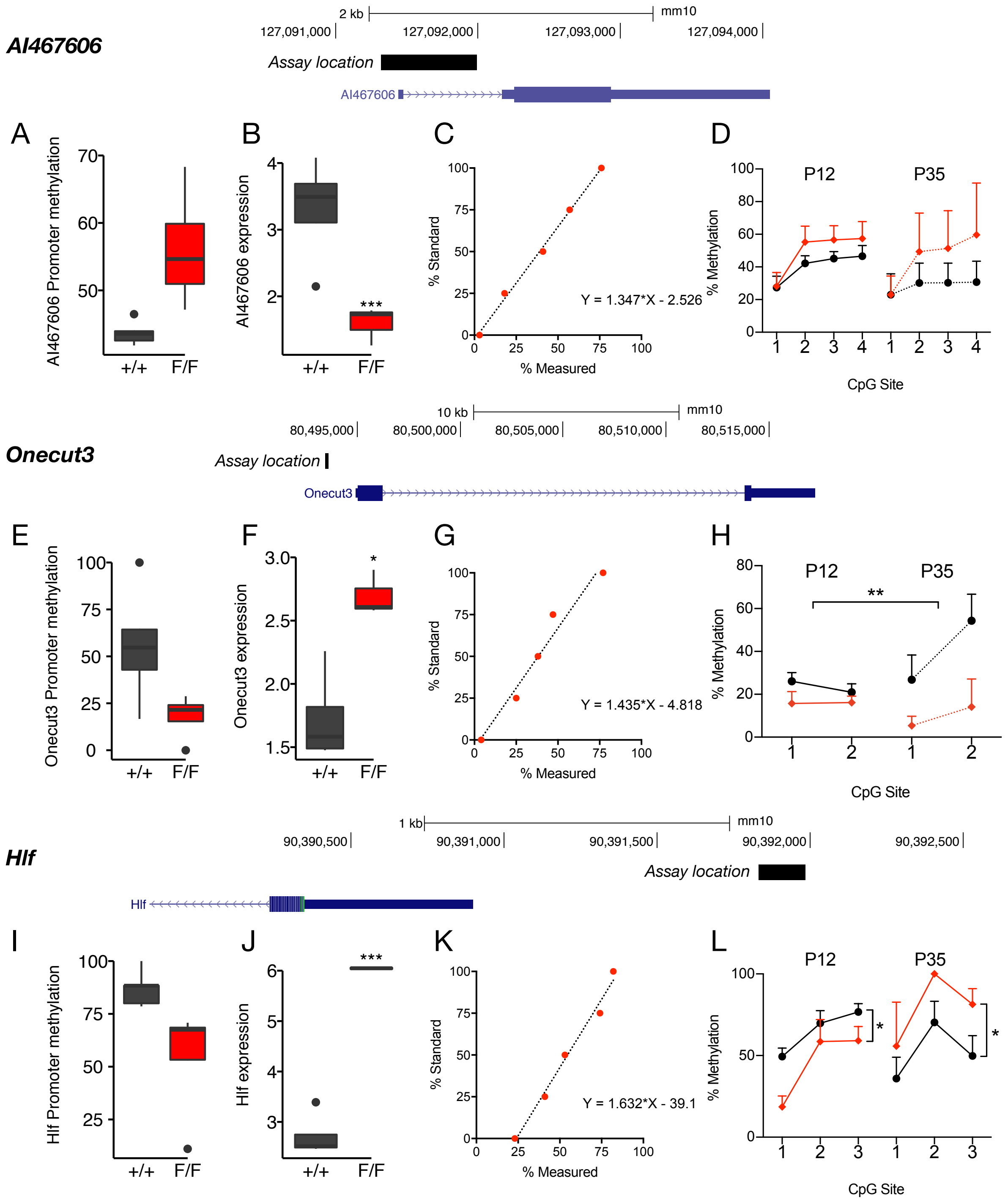

Supplementary Figure 3

Quantitative pyrosequencing of independent ARH neurons collected from P12 and P35 +/+ and F/F mice validates DMR analysis of WGBS. (A, E, I) Genes were selected from those with either hypo- or hypermethylated promoter DMRs. (B, F, J) Genes selected also exhibited either significantly up- or down-regulated expression. (C, G, K) Standard curves using mouse genomic DNA of known methylation state were used to validate linearity and accuracy of pyrosequencing assays. (D) Pyrosequencing analysis of AI467606 replicates directionality of differential methylation patterns observed in WGBS analysis, but does not reach significance (n=3-12). (H) Onecut3 shows replicates WGBS results (+/+-F/F=19.3% +/-6.09, df=34.5, p=0.0032) (n=3-12). (L) Hlf shows an interaction between age and genotype (F(1,26)=12.29, p=0.0017) with F/F mice showing decreased methylation at P12 (+/+-F/F=19.9% +/-8.71, df=30.7, p=0.03) and increased methylation at P35 (+/+-F/F=-27% +/-11.64, df=30.7, p=0.027) (n=3-12).

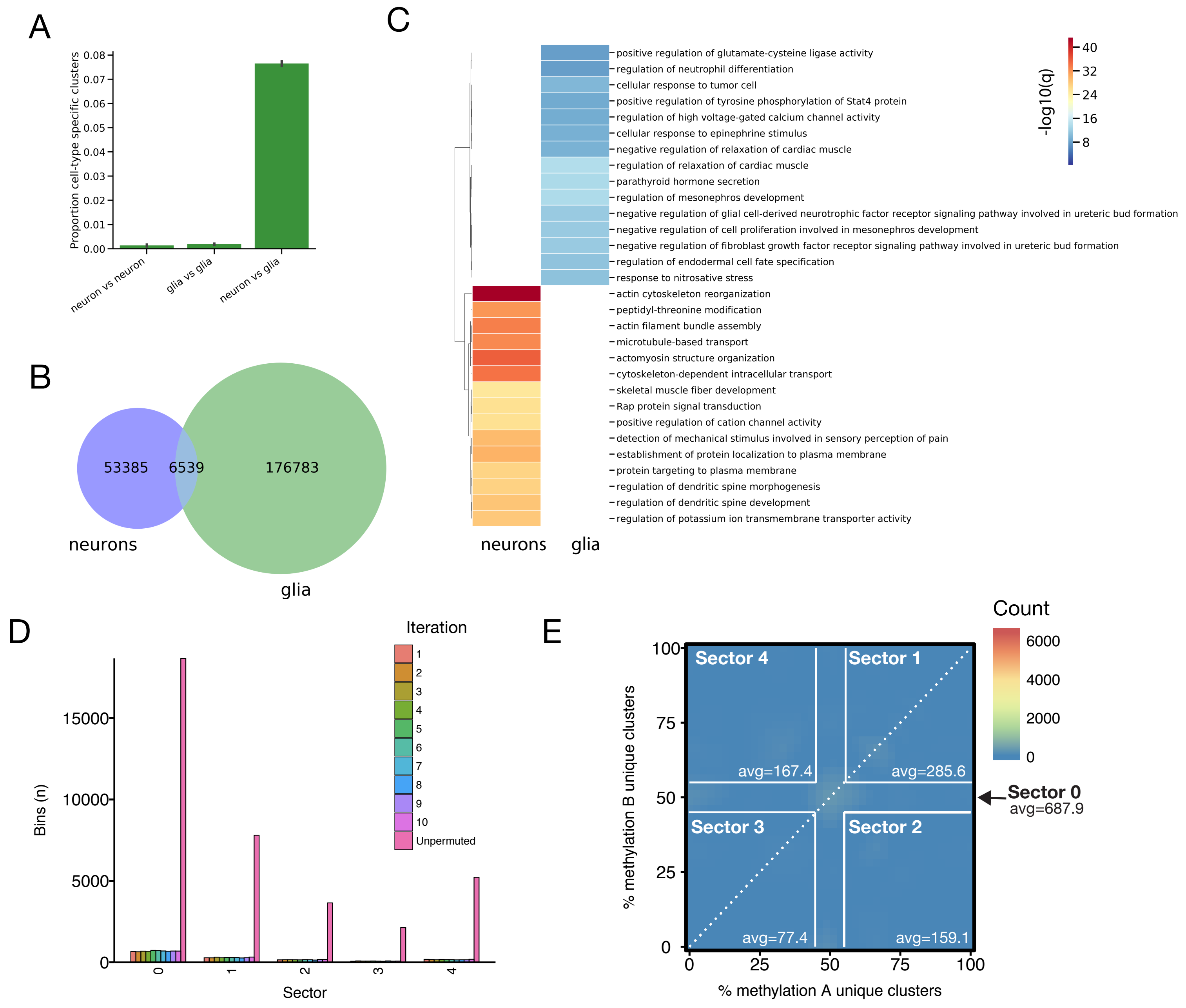

Supplementary Figure 4

Demonstration of read-level analysis on in-silico mixtures of WGBS data from FACS-sorted neurons and glia obtained from Lister et al., (2013). (A) Neuron and glia read-level methylation data from chromosome 19 was split into two halves and reads unique to either cell type were quantified in each split. When neuron- or glia-only datasets were split, read patterns unique to either split were extremely rare. In contrast, when comparing across cell type approximately 8% of read patterns are unique to one cell type or the other. This process was repeated 8 times to arrive at average proportions of cell type-specific read patterns. This indicates that read-level methylation patterns contain information unique to each cell type. (B) Venn diagram illustrating the number of bins containing either a neuron- or glia-specific read cluster. Very few bins contain read clusters unique to both cell types. (C) Top 15 GO biological process terms from GREAT analysis of bins containing neuron- and glia-specific read clusters. Color represents the -log10(q) value for each GO term. The preponderance of GO terms related to the expected cell type and lack of overlap supports the cell type-specificity of the identified regions. (D) Read-level analysis of randomly permuted read data results in substantially fewer unique bins compared to unpermuted data. (E) Two-dimensional density plot showing average density of unique bins in permuted data (averaged over 10 random permutations). Density scaling is identical to Figure 4B for illustrative purposes. False positive rates were 3.69%, 3.66%, 4.36%, 3.63%, and 3.2% for sectors 0, 1, 2, 3, and 4 respectively.

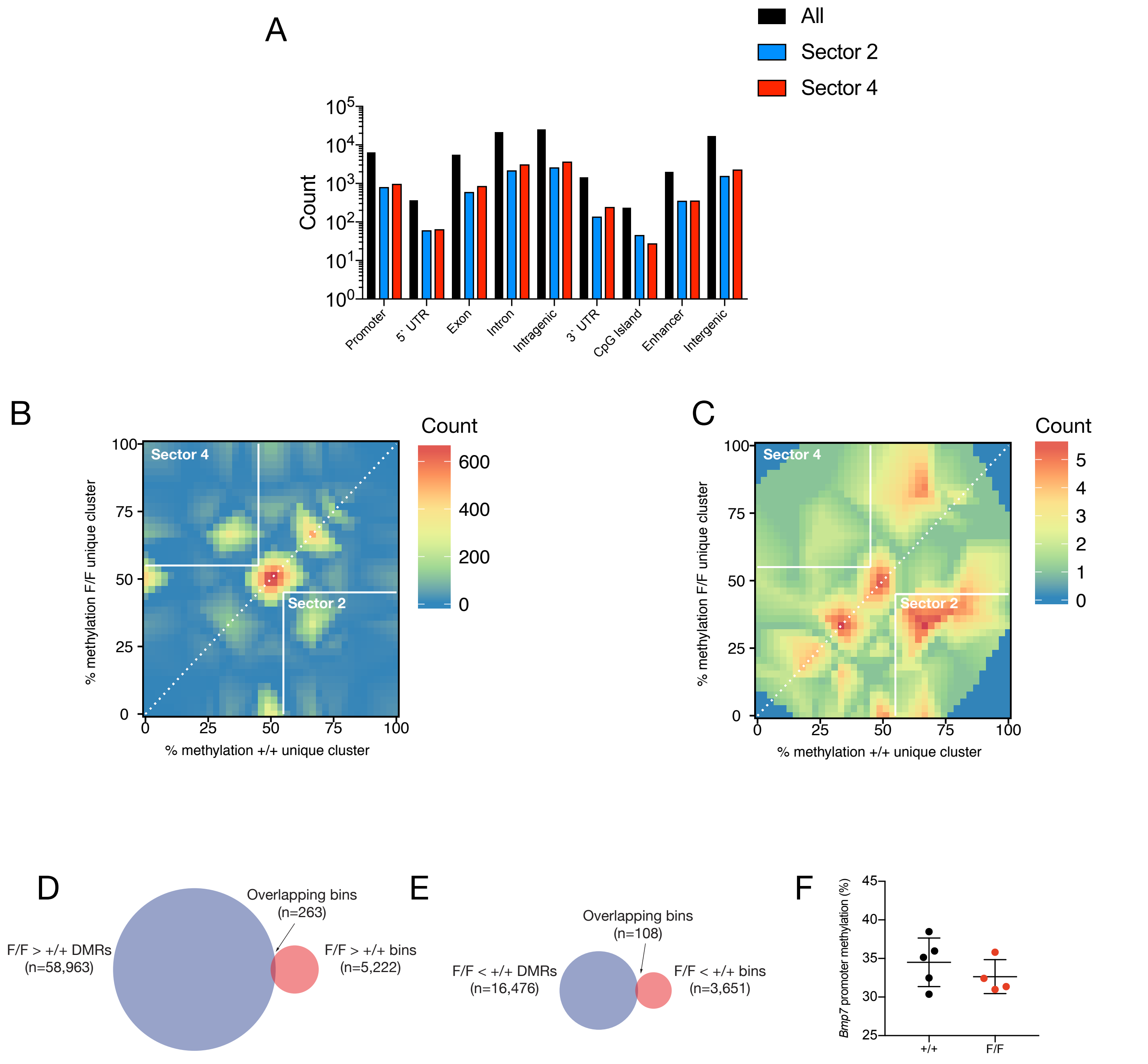

Supplementary Figure 5

(A) Counts of sector 2 and sector 4 bins located in defined genomic regions relative to counts of all four sectors combined. (B and C) Two-dimensional density plot showing relative methylation of F/F and +/+ unique read clusters and their respective methylation levels in promoter regions (B) and CpG islands (C). As described in Figure 4, Bins in sector 2 were defined by the simultaneous absence of hypermethylated (>= 55%) read clusters identified in +/+ mice and presence of novel hypomethylated (<= 45%) read clusters not present in +/+ mice. Bins in sector 4 were defined by the simultaneous presence of hypermethylated (>= 55%) read clusters identified in F/F mice and presence of novel hypomethylated (<= 45%) read clusters not present in F/F mice. (D) Venn diagram illustrating overlap between hypermethylated DMRs and sector 4 (F/F > +/+) bins. (E) Venn diagram illustrating overlap between hypomethylated DMRs and sector 2 (F/F < +/+) bins. (F) No difference in Bmp7 promoter methylation as measured by conventional per-CpG analysis.

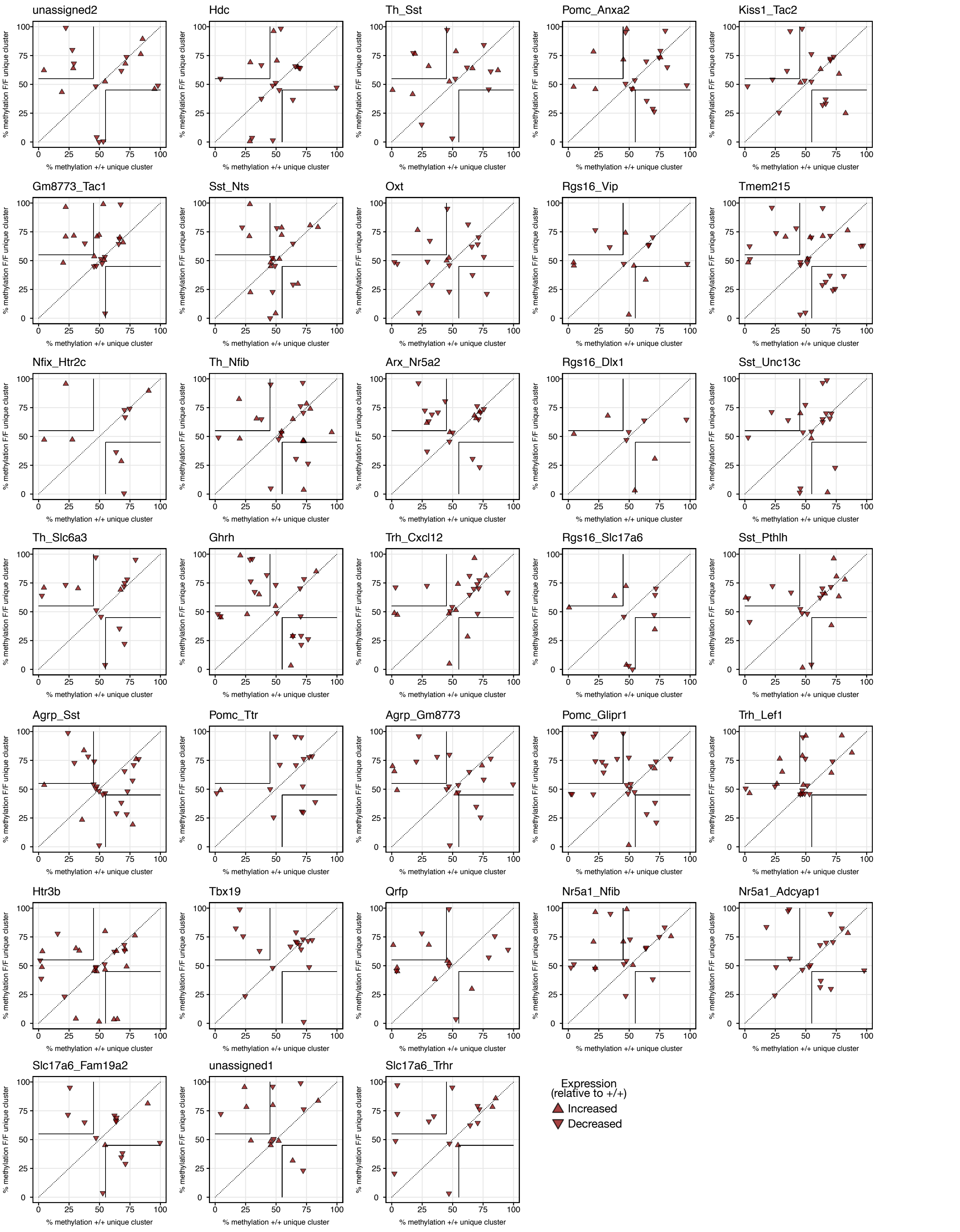

Supplementary Figure 6

Plots showing the location of promoter-associated bins in genes enriched in each of the neuronal cell types defined by Campbell et al. (2017).

ID	Genotype	NeuN+ nuclei	Final WGBS	Final RNA-Seq
A1	+/+	16910	X	Excluded (low RIN/low mapping)
A2	+/+	7267	X	X
A3	+/+	6508	X	X
A4	+/+	6390	X	X
A5	+/+	4492	X	X
B1	F/F	4646	Excluded (high duplicate rate/low coverage)	X
B2	F/F	9652	X	X
B3	F/F	11914	X	X
B4	F/F	11716	X	Excluded (low RIN/low mapping)
B5	F/F	10040	X	Excluded (low RIN/low mapping)

Supplementary table 1: Description of samples used and excluded in RNA-seq and WGBS experiments.

Region name	Avg. DMR length	N hypomethylated DMRs	N hypermethylated DMRs	Avg. hypomethylated DMR length	Avg. hypermethylated DMR length
Promoter	5000.00	3047	6098	216.74	214.82
3’ UTR	689.88	317	1332	213.71	214.16
5’ UTR	144.63	526	529	249.85	277.48
Exon	385.77	2002	5618	240.47	224.14
Intergenic	41388.16	8356	29783	232.74	224.72
Intragenic	24030.61	9101	31006	208.06	204.77
Intron	4389.04	7907	27870	209.07	207.32
CGI	655.02	689	502	261.13	259.15
Enhancer	1101.66	1924	1770	208.81	220.24

Supplementary table 2: Genomic location, count, and length statistics for DMRs (F/F-+/+).

Gene name	Assay location	Forward primer	Reverse primer	Sequencing primer
AI467606	chr7:127091319-127091987	GTTTTTTTTTGAGGTTAGTTT	TTTCAAAACCCTTCTTCC	AGTTTTTTAGGGTTTTTTAGAT
Onecut3	chr10:80493427-80493565	GGGAAAATGAGGAATAGAAA	CCAAAAATCTAATATCCACAATAC	AAAGGATTTGTGTAGTTT
Hlf	chr11:90391850-90392001	GTGTGGGGGAGAATATTT	ATCCCACATACTCCTCCA	TTTAGTTAGGAGAATTGAGA
Bmp7 (first assay)	chr2:172938700-172938800	TGGGGTTTTTTTAGTAATTTTTATAAGTAGA	AATCTAAAACAACTCCCCCTACAA	AGTAATTTTTATAAGTAGAATAAAAG
Bmp7 (second assay)	chr2:172938800-172938900	TGGGGGTTAGTTTGAGTAAGTT	AAAATTCCAACCAACCCAATAAA	GTTAGTTTGAGTAAGTTGAAT

Supplementary Table 3: PCR and sequencing primers for bisulfite pyrosequencing assays.
