# Supplementary material for: DNA methylation in AgRP neurons regulates voluntary exercise behavior in mice
Source: Nat Commun. 2019 Dec 2;10:5364. doi: 10.1038/s41467-019-13339-3 (PMC6889160; doi:10.1038/s41467-019-13339-3)
Supplement: Supplementary file 2 — Description of Additional Supplementary Files [file 41467_2019_13339_MOESM2_ESM.pdf]

Description of Additional Supplementary Files

File Name: Supplementary Data 1
Description: List of differentially methylated regions (DMRs) >10% difference identified by DSS

File Name: Supplementary Data 2
Description: RNA-Seq results (DESeq2)

File Name: Supplementary Data 3
Description: Location and methylation levels of 100bp bins identified by read-level analysis
