# Supplementary material for: DNA methylation in AgRP neurons regulates voluntary exercise behavior in mice
Source: Nat Commun. 2019 Dec 2;10:5364. doi: 10.1038/s41467-019-13339-3 (PMC6889160; doi:10.1038/s41467-019-13339-3)
Supplement: Supplementary file 6 — Reporting Summary [file 41467_2019_13339_MOESM6_ESM.pdf]

nature research

Corresponding author(s): Robert A Waterland (waterland@bcm.edu)

Last updated by author(s): Oct 3, 2019

Reporting Summary

Nature Research wishes to improve the reproducibility of the work that we publish. This form provides structure for consistency and transparency in reporting. For further information on Nature Research policies, see Authors & Referees and the Editorial Policy Checklist.

Statistics

For all statistical analyses, confirm that the following items are present in the figure legend, table legend, main text, or Methods section.

n/a	Confirmed	
☐	☒	The exact sample size (*n*) for each experimental group/condition, given as a discrete number and unit of measurement
☐	☒	A statement on whether measurements were taken from distinct samples or whether the same sample was measured repeatedly
☐	☒	The statistical test(s) used AND whether they are one- or two-sided<br>*Only common tests should be described solely by name; describe more complex techniques in the Methods section.*
☐	☒	A description of all covariates tested
☐	☒	A description of any assumptions or corrections, such as tests of normality and adjustment for multiple comparisons
☐	☒	A full description of the statistical parameters including central tendency (e.g. means) or other basic estimates (e.g. regression coefficient) AND variation (e.g. standard deviation) or associated estimates of uncertainty (e.g. confidence intervals)
☐	☒	For null hypothesis testing, the test statistic (e.g. *F*, *t*, *r*) with confidence intervals, effect sizes, degrees of freedom and *P* value noted<br>*Give P values as exact values whenever suitable.*
☒	☐	For Bayesian analysis, information on the choice of priors and Markov chain Monte Carlo settings
☒	☐	For hierarchical and complex designs, identification of the appropriate level for tests and full reporting of outcomes
☒	☐	Estimates of effect sizes (e.g. Cohen's *d*, Pearson's *r*), indicating how they were calculated

Our web collection on statistics for biologists contains articles on many of the points above.

Software and code

Policy information about availability of computer code

Data collection	*Provide a description of all commercial, open source and custom code used to collect the data in this study, specifying the version used OR state that no software was used.*
Data analysis	Statistical analysis: SPSS 17, R 3.4<br>Trimming: TrimGalore 0.4.4<br>Read QC: FastQC 0.11.5<br>RNA-Seq alignment: Hisat2 2.1.0<br>Read annotation: featureCounts 1.5.3<br>Differential expression analysis: DESeq2 1.16.1<br>WGBS alignment: Bismark 0.18.1<br>Removal of duplicates: Picard 2.10.10<br>Coordinate sorting: SAMtools 1.9<br>DMR analysis: DSS 3.7<br>Image analysis: ImageJ 1.51g<br>DMR annotation: GREAT 3.0.0

For manuscripts utilizing custom algorithms or software that are central to the research but not yet described in published literature, software must be made available to editors/reviewers. We strongly encourage code deposition in a community repository (e.g. GitHub). See the Nature Research guidelines for submitting code & software for further information.

Data

Policy information about availability of data

All manuscripts must include a data availability statement. This statement should provide the following information, where applicable:

- Accession codes, unique identifiers, or web links for publicly available datasets
- A list of figures that have associated raw data
- A description of any restrictions on data availability

Raw data has been deposited in GEO under accession number GSE122405

Field-specific reporting

Please select the one below that is the best fit for your research. If you are not sure, read the appropriate sections before making your selection.

☒ Life sciences ☐ Behavioural & social sciences ☐ Ecological, evolutionary & environmental sciences

For a reference copy of the document with all sections, see nature.com/documents/nr-reporting-summary-flat.pdf

Life sciences study design

All studies must disclose on these points even when the disclosure is negative.

Sample size	Sample sizes for body weight, metabolic, and histology studies were determined based on previously published experiments of similar design (MacKay et al., 2017, Baker et al., 2015). RNA-Seq and WGBS experiments were conducted with n=5 libraries per genotype with one dissection per library.
Data exclusions	WGBS and RNA-Seq libraries were excluded if they did not pass quality control measures. A total of 3 libraries were excluded from RNA-Seq analysis due to low RIN values (<5) and/or low mapping rates (<30%). One library was excluded from WGBS due to having substantially lower coverage (1.5x vs. 10x average coverage in remaining libraries) and higher duplication levels (85% vs. ~45% for remaining libraries).
Replication	Body weight and metabolic studies were carried out in two cohorts of mice consuming either standard diet or high-fat diet. Running wheel studies were conducted using two separate cohorts of mice. Select DMRs identified by WGBS were validated in an independent cohort at two timepoints using bisulfite pyrosequencing.
Randomization	Samples were randomized at all experimental stages, and animal manipulations were balanced across sex/genotype where appropriate.
Blinding	Animal IDs, sample and slide labels were anonymized to conceal sex, age, and genotype. All molecular and histological analysis was performed by an experimenter blind to these factors.

Reporting for specific materials, systems and methods

We require information from authors about some types of materials, experimental systems and methods used in many studies. Here, indicate whether each material, system or method listed is relevant to your study. If you are not sure if a list item applies to your research, read the appropriate section before selecting a response.

Materials & experimental systems

n/a	Involved in the study	
☐	☒	Antibodies
☒	☐	Eukaryotic cell lines
☒	☐	Palaeontology
☐	☒	Animals and other organisms
☒	☐	Human research participants
☒	☐	Clinical data

Methods

n/a	Involved in the study	
☒	☐	ChIP-seq
☐	☒	Flow cytometry
☒	☐	MRI-based neuroimaging

Antibodies

Antibodies used	RFP (Abcam ab34771, 1:1000)<br>DNMT3A (Abcam ab2850, 1:1000)<br>5-mC (Cell Signaling Technology 28692, 1:1000)<br>5-hmC (Abcam ab214728, 1:1000)<br>NeuN (Millipore ABN78, 1:4000)
Validation	RFP: Affinity purified, validated by ELISA, no primary control<br>DNMT3A: Affinity purified, validated by ELISA, no primary control

5-mC: Affinity purified, validated by ELISA, dot blot, and MeDIP, no primary control
5-mC: Affinity purified, validated by ELISA, dot blot, and MeDIP, no primary control
NeuN: Affinity purified, validated by Western blot, no primary control

Animals and other organisms

Policy information about studies involving animals; ARRIVE guidelines recommended for reporting animal research

Laboratory animals	NPY-hfGFP mice (Jackson Laboratory), AgRP-IRES-Cre; Dnmt3a+/+ and AgRP-IRES-Cre; Dnmt3aF/F mice, Ai34(RCL-Syp/tdT)-D (Synaptophysin-TdTomato mice)
Wild animals	*Provide details on animals observed in or captured in the field; report species, sex and age where possible. Describe how animals were caught and transported and what happened to captive animals after the study (if killed, explain why and describe method; if released, say where and when) OR state that the study did not involve wild animals.*
Field-collected samples	*For laboratory work with field-collected samples, describe all relevant parameters such as housing, maintenance, temperature, photoperiod and end-of-experiment protocol OR state that the study did not involve samples collected from the field.*
Ethics oversight	The protocol was approved by the Institutional Animal Care and Use Committee of Baylor College of Medicine

Note that full information on the approval of the study protocol must also be provided in the manuscript.

Flow Cytometry

Plots

Confirm that:

☒ The axis labels state the marker and fluorochrome used (e.g. CD4-FITC).

☒ The axis scales are clearly visible. Include numbers along axes only for bottom left plot of group (a 'group' is an analysis of identical markers).

☒ All plots are contour plots with outliers or pseudocolor plots.

☒ A numerical value for number of cells or percentage (with statistics) is provided.

Methodology

Sample preparation	Microdissected arcuate nuclei were purified by ultracentrifugation against a 1.8M sucrose column at 100k rcf. Purified nuclei were labelled using rabbit anti-NeuN (Millipore ABN78; 1:4000) followed by Alexa488-conjugated goat anti-rabbit IgG (ThermoFisher).
Instrument	Sony SH800
Software	Sony FACS sorting software
Cell population abundance	NeuN+ and - nuclei comprised between 7-11% of total event counts (1:0.81 NeuN+). An average of 10927.54 (+/-2023.51) NeuN + nuclei were collected per sample.
Gating strategy	FSC and SSC gates were established to eliminate debris. TO-PRO-3 labeling was used to establish a minimal fluorescent value to delineate nuclear material and remove doublets. A histogram on the Alexa488 channel was used to establish gates for NeuN labeling.

☒ Tick this box to confirm that a figure exemplifying the gating strategy is provided in the Supplementary Information.
